# Modelling symbols among the first-sedentary communities: Emergence of clay figurines in Epipaleolithic Southwest Asia

**DOI:** 10.1371/journal.pone.0360388

**Published:** 2026-11-11

**Authors:** Laurent Davin, Marion Sindel, Mina Weinstein-Evron, Reuven Yeshurun, Daniel Kaufman, Leore Grosman, Anna Belfer-Cohen, Hamoudi Khalaily, François R. Valla

**Affiliations:** 1 The Hebrew University of Jerusalem, Institute of Archaeology, Jerusalem, Israel; 2 CNRS. UMR 8068 Technologie et Ethnologie des Mondes PréhistoriqueS (TEMPS), Nanterre, France; 3 CNRS. UAR 3132 Centre de recherche français à Jérusalem (CRFJ), Jerusalem, Israel; 4 Israel Antiquities Authority (IAA), Jerusalem, Israel; 5 University of Haifa, School of Archaeology and Maritime Cultures, Zinman Institute of Archaeology, Haifa, Israel; Universita degli Studi di Ferrara, ITALY

## Abstract

The emergence of clay technologies and their use in symbolic practices marks a momentous techno-symbolic innovation for human societies. While in North Africa, Europe and East Asia, this innovation was sporadically attested during the Upper Palaeolithic, in Southwest Asia, it has been commonly believed to emerge in full much later, as one of the innovations associated with Neolithic agricultural societies. Here, shifting back the ‘revolution of symbols’, we report evidence of the earliest clay figurative tradition in Southwest Asia with the discovery of 154 figurative artefacts from three Natufian (15,000–11,650 years cal. BP) sites in the Southern Levant (Israel). Through a combined multidisciplinary approach, we demonstrate that children and adults manufactured these objects to produce new symbols with new figurative techniques and practices used as their primary media of figurative expression. Our study provides important original insights regarding the demographics of craft production and the development of symbolic practices at the beginning of sedentism that influenced the Neolithic transition in Southwest Asia.

## Introduction

The scarcity of symbolic material expressions in the later prehistory of Southwest Asia has long been noticed [1], and indeed, the archaeological record prior to the emergence of the Natufian culture in the Levant (Late Epipalaeolithic: 15,000–11,650 cal. BP) is poor in this regard. Examples have remained scarce despite the recent widespread and thorough excavation of Upper Palaeolithic (c. 47,000–24,000 cal. BP) and Early/Middle Epipalaeolithic (c. 24,000–15,000 cal. BP) sites. The pre-Natufian techno-symbolic productions mainly include a limited repertoire of personal ornaments made of hard animal tissues [e.g., 2], mineral pigments [e.g., 3], two incised zoomorphic figurations on limestone slabs [4, 5], as well as rare bone tools and stones incised with non-figurative decorations (geometric and abstract motifs) [5–12].

The materialisation of symbolic expression changes abruptly in the Natufian culture, which marks the transition from hunter-gatherer Palaeolithic societies into fully-fledged agricultural economies of the Neolithic [for details see 13–15]. Natufian communities were the first hunter-fisher-gatherers in Southwest Asia to adopt a sedentary lifestyle, a dramatic economic revolution (‘broad-spectrum revolution’ [16],) and societal change associated with growing social complexity [17]. This was reflected in various aspects of their rich and diverse material culture and practices, e.g., durable stone-built architecture [18–21], graveyards and complex funerary practices [e.g., 22–24], symbolic manifestations [25–27], extensive repertoire of personal ornaments [28–31], mineral and organic colorants [32, 33], sound instruments [34], shamanism [35] and feasting [36]. These first sedentary communities, in transition to new concepts of relationships with their social and natural environments [17], expanded pre-existing non-figurative expressions [37]. On the other hand, in a break with the past, these communities produced new figurative, three-dimensional, durable and tangible expressions: portable anthropomorphic (Fig 1 Aa,O-X), phallic (Fig 1Q) and zoomorphic (Fig 1 Ab-N,X) representations. These naturalistic or schematic figurines were recovered from open-air and cave sites mainly in Mount Carmel, the Galilee, and the Jordan Valley (Fig 2A-B), where they were manufactured from ungulate bone and stone (limestone, calcite, flint nodule, basalt) throughout the three millennia of Natufian existence [25,50,51]. Although some figurations were incorporated into functional objects, such as bone sickle handles (Fig 1B-C), stone pestles [e.g., 40] or personal ornaments such as ornithomorphic bone pendants (Fig 1I), most of them were autonomous, non-functional (non-mundane functions) carved objects.

**Fig 1:**
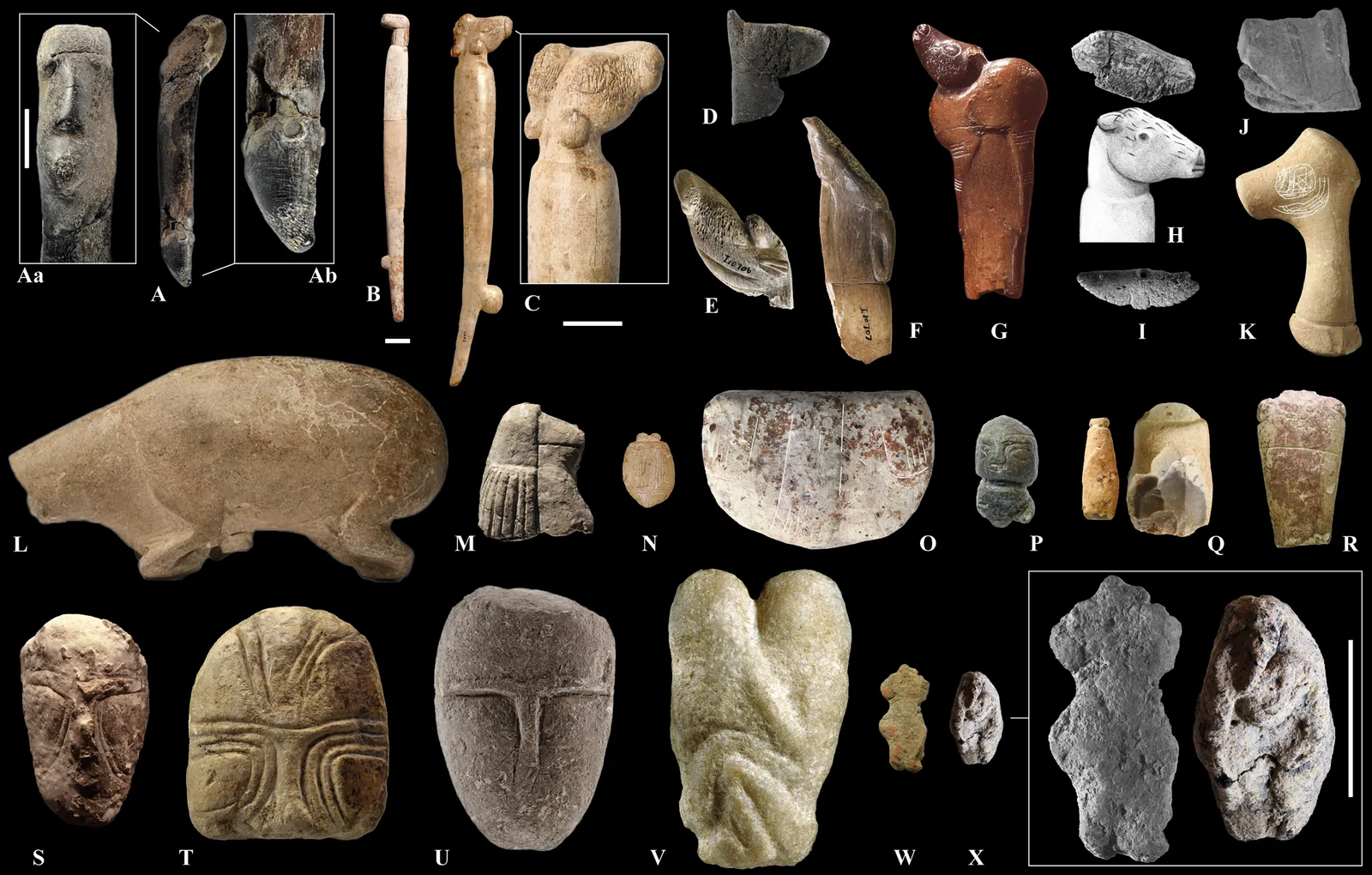
Sample of Natufian zoomorphic (Ab-N,X), anthropomorphic (Aa,O-X) and phallic (Q) bone (A-I), stone (J-V) and clay (W-X) figurations from the Southern Levant: (A,D,K,M) Nahal Oren – LN [38]; (B,C,E,F,O) Kebara – EN [39]; (G,P,Q) el-Wad – EN-LN [23]; (H-J) Wadi Hammeh 27 – EN [40,41] courtesy of P. Edwards); (L) Umm ez-Zuweitina – EN [42]; (N,R-T) Eynan-Mallaha – EN-FN [19,43,44]; (U,X) Nahal Ein Gev II – LN [45, 46]; (V) Wadi Khareitoun – EN-LN [47]; (W) Hayonim Terrace – LN [48]. Natufian periods: Early (EN), Late (LN), Final (FN). Scales 2 cm. (Photos L.D., (S-T,W-X) The Israel museum Jerusalem, (A-D,G,K-M,P) Israel Antiquities Authority, (V) The Trustees of the British Museum, (H-J) P. Edwards).

**Fig 2:**
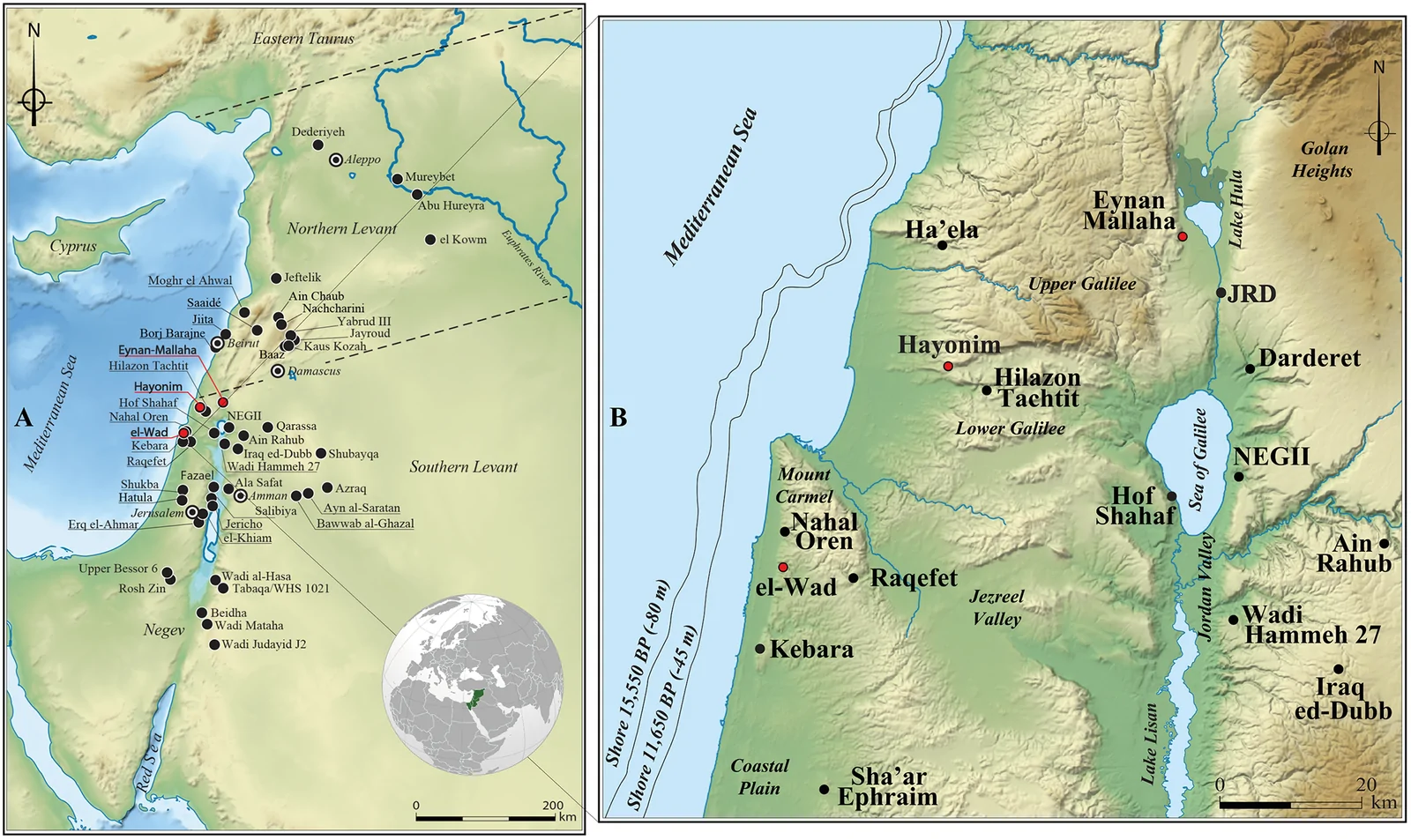
Map of the distribution of Natufian sites in, A: the Levant; B: the region of el-Wad, Hayonim and Eynan-Mallaha in the Southern Levant with the position of the Mediterranean shoreline at the beginning and the end of the Natufian (Sea level at -80m and -45m [49]) and of the Lake Lisan at 15,000 BP. (CAD L.D. Background map republished from Wikipedia under a CC BY license, with permission from Ivan d’Hostingue and NordNordWest, original copyright 2023).

Despite this richness in Natufian figurative expressions, only two figurines modelled in clay have thus far been identified in the entire Natufian repertoire (Fig 1 WX). These small anthropomorphic figurines, both dated to the Late Natufian, were discovered in Hayonim Terrace (Fig 1W) [48,52,53] and Nahal Ein Gev II (Fig 1X) [46] respectively (Fig 2A-B and S1 Table). The former has been interpreted as a stylised female representation given its secondary sexual characteristics (accentuated hips) [48,52,53], while the latter depicts an interaction between a woman and a goose [46]. These two clay figurines are the only sexed Natufian anthropomorphic representations known so far. The analysis of the figurine from Hayonim Terrace (Fig 1W) showed that it had been modelled by pinching and smoothing of a reddish clay, which was later placed near and/or in contact with fire [54,55]. The transformation of the figurine from Nahal Ein Gev II (Fig 1X) was more complex as it involved the meticulous use of several tools and techniques to produce a play of light and shadow, emphasising perspective. The figurine was later colored red with hematite and baked at low temperature (≤400°C) [46].

In addition, of all the pre-Neolithic contexts from Southwest Asia (Anatolia, Levant, Mesopotamia, and Zagros), only seven other clay objects, dated to the Late Epipalaeolithic, have been interpreted as belonging to the symbolic sphere (S1 Table and Fig 3). These small artefacts have been modelled to create diverse geometric shapes (unrelated to the bone or stone figurations) that do not, at first glance, suggest an anthropomorphic or zoomorphic representation.

**Fig 3:**
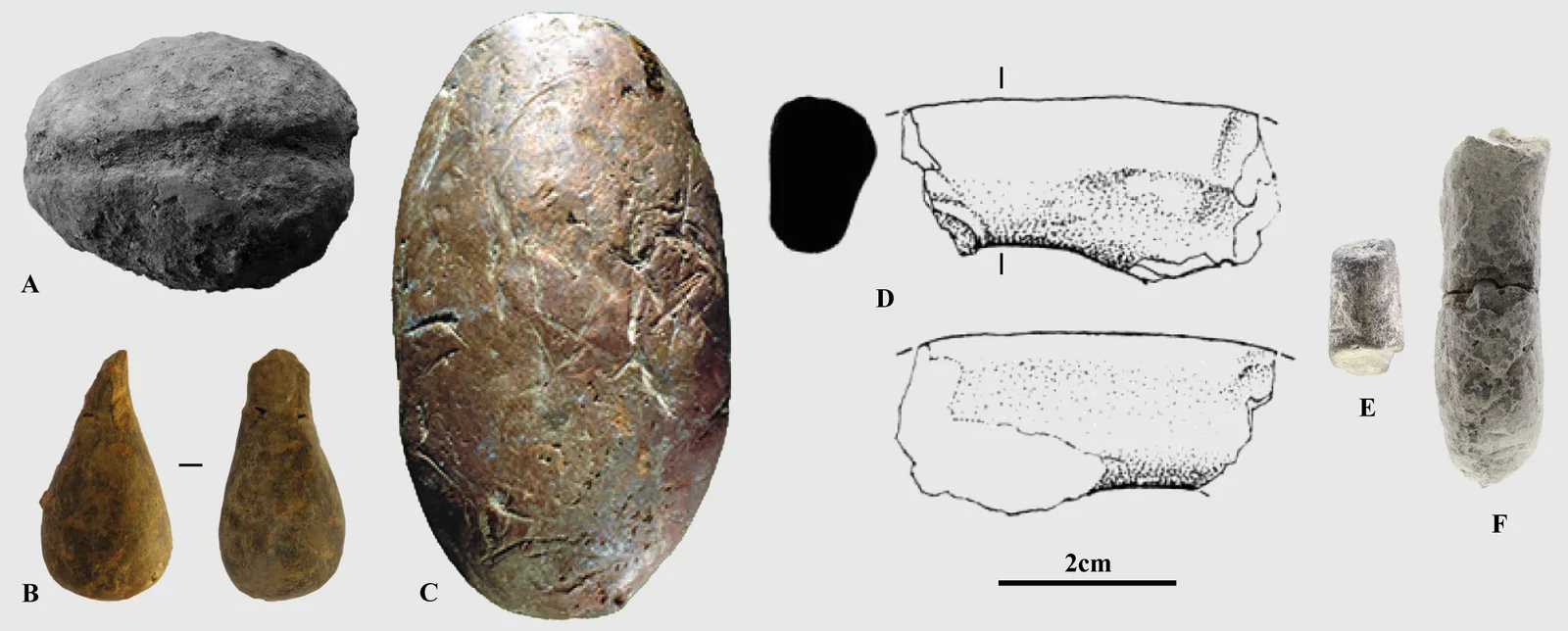
Late Epipaleolithic clay artefacts from the Levant and Anatolia (A) Moghr el-Ahwal – EN ([56], courtesy of A. Garrard); (B) Direkli Cave ([57], courtesy of C.M. Erek); (C) Hayonim Cave – EN [14]; (D) Gilgal II – LN [58]; (E-F) Salibiya I – LN [55] (Photos L.D.). Natufian periods: Early (EN), Late (LN).

Given the meagre evidence of the figurative use of clay in the Epipalaeolithic of Southwest Asia, it has been considered in the current state of research that these few pre-Neolithic clay artefacts were only rare and rudimentary first experiments of a clay figurative tradition that would emerge in full from the Early Neolithic onwards [55,58–60]. In the hunter-cultivator villages of the Pre-Pottery Neolithic A (PPNA) (c. 11,650–10,600 cal. BP) the clay figuration consists of rare and exclusively anthropomorphic figurines (n < 15) [59, 60] and their cylindrical fragments (body parts and appendages) [55,58]. Such items were reported from the Zagros [61], the Northern Levant (Mureybet – [62]) and mainly from the Southern Levant, e.g., Gilgal I [58], Netiv Hagdud [63], Jericho [64], Dhra‘ [65] and el-Wad [66]. These figurations, baked or not, are primarily stylised and small (<5cm) with a “pillared” (or stalk-like) upper body, most of them exhibiting female secondary sexual characteristics (breasts, hips) in a standing or frequently seated position (see [60] for a synthesis). This tradition, still variable in the heat treatment of the clay [59], evolved through time as regards quantity (thousands of artefacts) and typological variability (anthropomorphic, zoomorphic and hybrid), becoming the primary medium of expression for portable figuration of the fully-fledged agricultural communities during the Pre-Pottery Neolithic B (PPNB) (c. 10,500-9,000 cal. BP) [60,67,68] through the later emergence of pottery in the 7^th^ millennium BCE [69]. According to this paradigm, the emergence of figurative use of clay would go hand in hand with the emergence of the Neolithic productive economy [58]. This tremendous change in lifeways would have led to a shift in the use of imagery, reflecting the struggle to control new structures of economy, social organisation, and symbolism [68], that Jacques Cauvin [62] identified as the ‘revolution of symbols’, the symbolic, cognitive and ontological foundations of the Neolithic transition. Thus, contrary to what has been observed in Upper Palaeolithic contexts in Europe [e.g., 70–73], in North Africa [e.g., 74,75] or in East Asia [e.g., 76,77], the figurative use of clay would not be apparent in pre-Neolithic hunter-gatherers of Southwest Asia.

To challenge this long-standing paradigm and the socio-economic crucible of the ‘revolution of symbols’, we have sought to uncover new evidence of using clay for figurative purposes in the first sedentary communities of the Southern Levant. Excavations at Natufian sites of el-Wad Terrace (EWT hereafter) in the Mount Carmel, Hayonim Terrace (HT hereafter) in the Galilee and Eynan-Mallaha (EM hereafter) in the Upper Jordan Valley (Fig 2; see a detailed presentation in the Materials and Methods section), followed by a meticulous sorting of the sieved sediments from specific contexts encompassing the whole duration of the Natufian culture, yielded tens of thousands of small (mostly less than 2 cm^3^) heated clay and sediment lumps (c. 40 kg in total) of different shapes and colors (S2 Table and S1 Fig). These Natufian clay lump collections, which had never been investigated before, were believed to be related to the manufacture of mineral pigments by the heat treatment of rich iron clays [78]. Their thorough re-evaluation led to the discovery of over a thousand artefacts, evidence for the earliest clay ornamental [31] and figurative traditions in Southwest Asia together with clay shapings (geometric shapes, non-diagnostic fragments and non-figurative pellets) related to manipulation of clay. In this preliminary study of the Natufian clay figurative tradition, we report on the identification of 15 ornithomorphic, zoomorphic and anthropomorphic figurines (Fig 4; Table 1; S4-18 Figs) as well as 139 ‘cylinders’ representing most probably fragments of body and appendages (Fig 5; S3 Table; S18-S24 Figs). This collection covers the three phases of the Natufian culture (Early: c. 15−13 ka cal. BP; Late: c. 13–11.65 ka cal. BP; Final: c. 12.5–11.65 ka cal. BP), enabling to follow the spatiotemporal evolution of the clay techno-symbolic practices in three major settlements of the Southern Levant. Through their technological, use-wear, archaeometric and dermatoglyphic analysis, we provide important original insights into the demographics of craft production and the interaction between symbolic expression and childhood. We redefine the dialectical relationships between the chrono-cultural, socio-economic and techno-symbolic developments at the crossroads of Sedentarisation and Neolithisation processes in Southwest Asia.

**Table 1:** List and morphometric measurements of the Natufian clay figurines from Hayonim Terrace (HT) and Eynan-Mallaha (EM) included in this study.

N° on Fig 4	Site	Inventory	Context	Natufian Phase	Figuration Type	Length (mm)	Width (mm)	Depth (mm)	Weight (gr.)	Burnt (Yes/No)
A	EM	EM97 6140	Shelter 215–228	Final	Zoomorphic	16	11,04	7,16	0,91	Y
B	EM	EM99 7121	Stony Layer	Final	Zoomorphic	12,77	10,24	9,47	0,51	N
C	EM	EM75 4551.17	Shelter 131	Early	Anthropomorphic	15,16	10,47	8,63	1,17	N
D	EM	EM01 8745	Shelter 228	Final	Anthropomorphic	21,36	18,34	10,95	2,59	Y
E	EM	EM97 6143	Shelter 215	Final	Ornithomorphic	27,48	20,37	12,4	4,94	Y
F	EM	EM75 4585	Shelter 131	Early	Ornithomorphic	13,74	7,08	5,65	0,45	N
G	EM	EM05 9803	Shelter 202	Final	Ornithomorphic	7,92	4,3	4,12	0,16	Y
H	EM	EM76 4562	Shelter 131	Early	Ornithomorphic	20,55	9,05	6,66	0,6	Y
I	EM	EM74 3399	Shelter 51	Early	Ornithomorphic**?**	24,17	11,04	7,79	1,8	N
J	EM	EM74 3339	Shelter 131	Early	Ornithomorphic	9,95	10,6	7,74	0,52	N
K	EM	EM98 6600	Stony Layer	Final	Ornithomorphic	12,41	6,34	4,94	0,26	Y
L	EM	EM97 5828	Shelter 215–228	Final	Ornithomorphic	9,79	5,94	5,51	0,28	Y
M	HT	HT86 1632	Outside	Late	Ornithomorphic	15,04	10,71	8,29	0,72	Y
N	HT	HT86 1272	Outside	Late	Ornithomorphic	10,25	6,91	7,07	0,35	N
O	EM	EM97 6104	Shelter 215–228	Final	Ornithomorphic	11,05	6,34	5,37	0,33	Y
					**Mean**	15,18	9,92	7,45	1,04	
					**Stand. Dev**	5,77	4,43	2,28	1,26	

**Fig 4:**
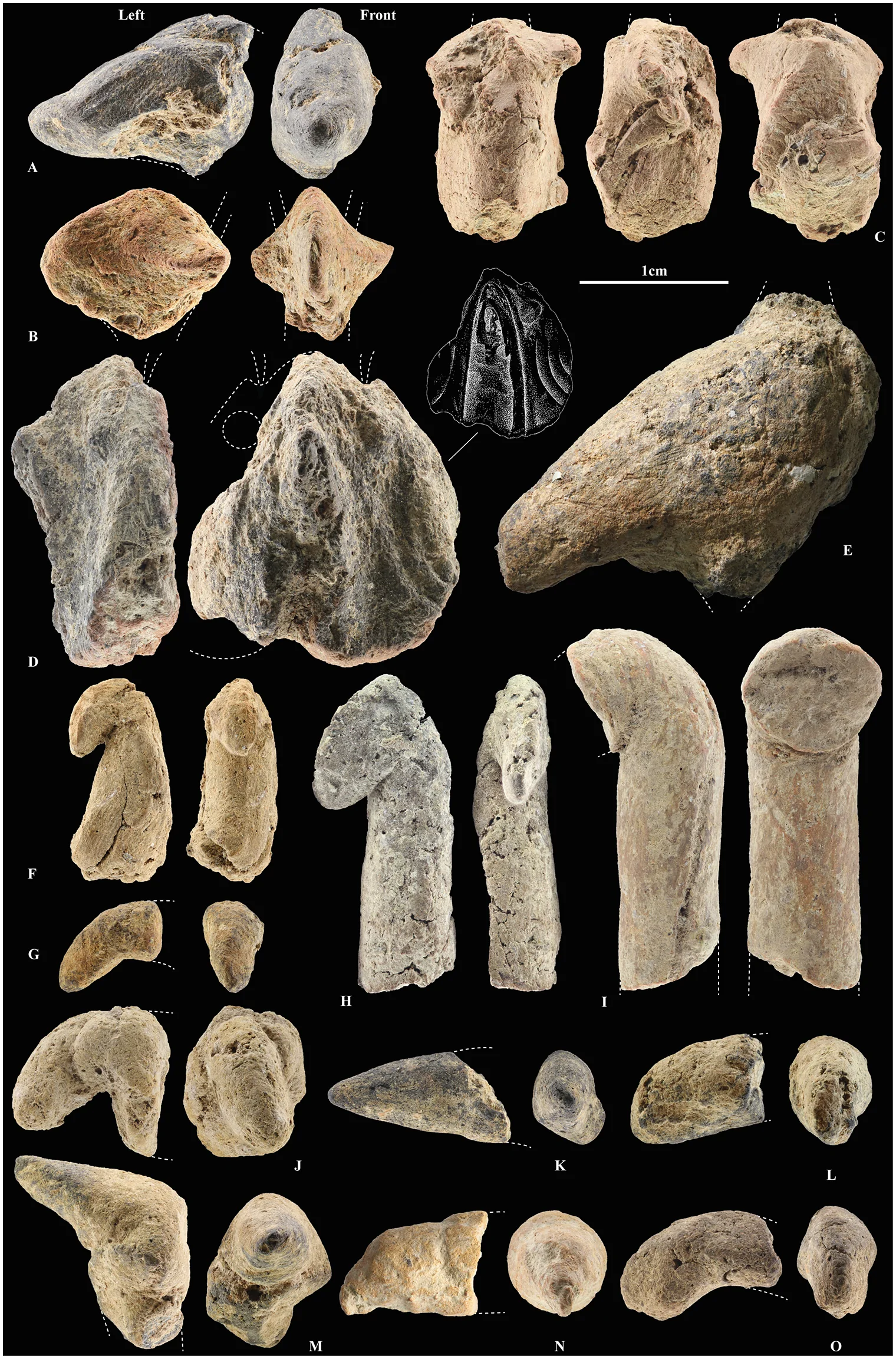
Fragments of zoomorphic (A-B), anthropomorphic (C-D) and ornithomorphic (E-O) clay figurines from the Natufian (Early to Final) of EM (A-O) and HT (M-N) (Photos and CAD L.D.). (Drawing Artist Credit: Dasha Lokshin, Haifa University).

**Fig 5:**
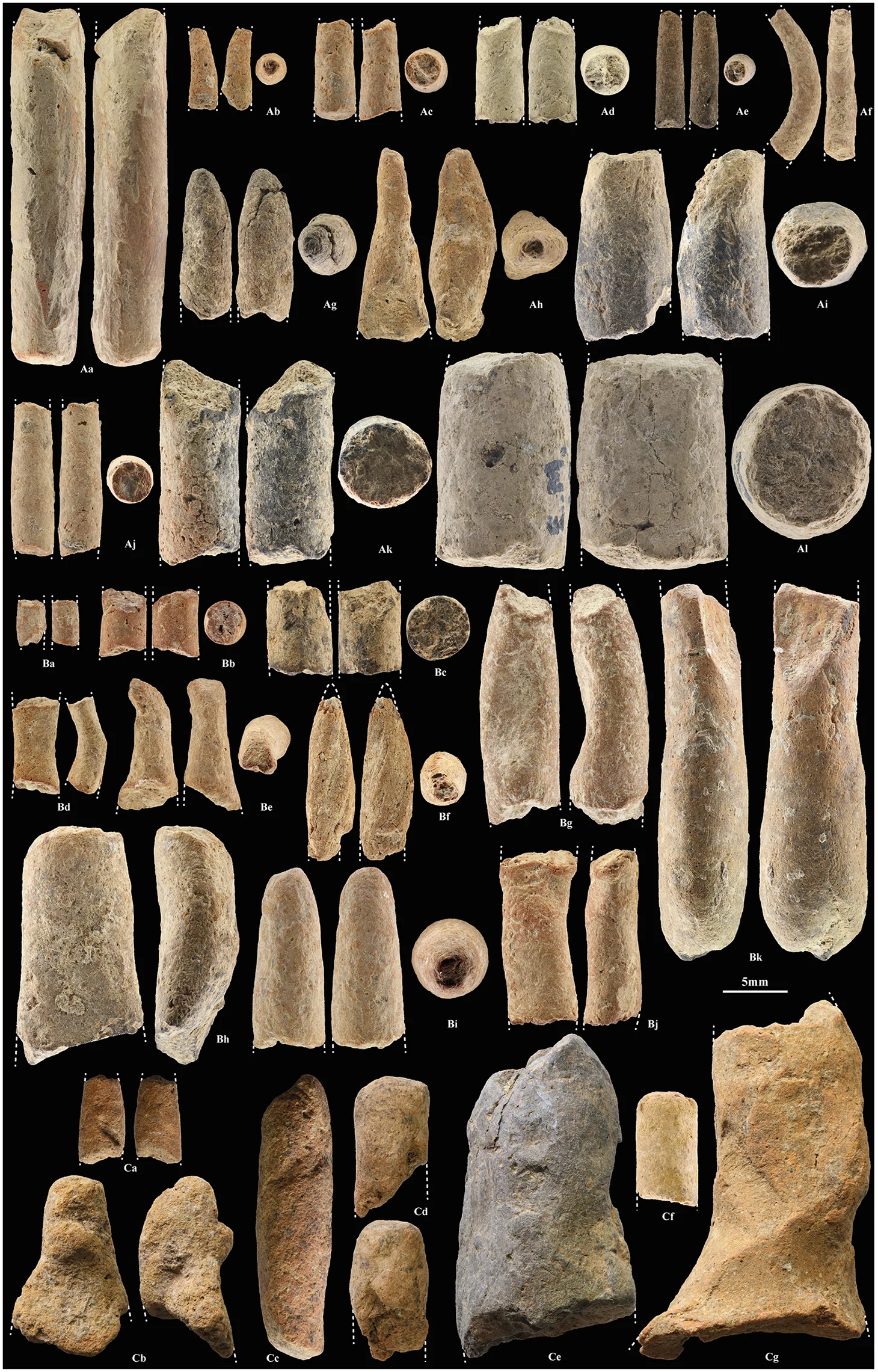
Sample of Natufian (Early to Final) clay cylinders from EM (A), HT (B) and EWT (C). (Photos and CAD L.D.).

## Results

### State of completeness

All the contexts in which these artefacts were discovered were checked to ensure that they were indeed Natufian, free of admixture from later occupations. None of the elements presented here are naturally occurring lumps of clay modelled accidentally. These are all objects manipulated by humans, as evidenced by fingertip-depressions, palm prints and fingerprints, and intentionally modelled, as evidenced by various technical marks (incisions, imprints, grooving, smoothing, inserting). Only two artefacts presented here, an ornithomorph figurine from Early Natufian EM (Fig 4F) and a cylinder from Early Natufian EWT (S18E Fig), were discovered complete. All the others are fragments, more or less close to their original form, of which fracture surfaces suggest their real extent (see photos with dotted lines for which the explanation is given in the individual description of the artefacts). This high fragmentation rate, probably linked primarily to the fragility of these small artefacts, is similar to that observed in the assemblages discovered in the Upper Palaeolithic Pavlovian of Europe (c. 32,000–27,000 cal. BP [70]. The figurines are mainly fragments of heads (Fig 4A-B,G,J-O) that we have identified thanks to the regularity of their surface treatment, their coloring and the symmetry of their profile modeling. In the case of mammal heads (Fig 4A-B), their identification as such relies also on a stylistic comparison with schematic Natufian mammal figurations made of bone and stone (Fig 1A,B,D,K). The identification of fragments representing bird heads and beaks (Fig 4G,J-O), is also based on a stylistic comparison with contemporaneous less fragmentary ornithomorphic figurations (Fig 1X and Fig 4F,H) [46] and similar PPNA figurations [e.g., 79] (S25 Fig).

### Raw material selection and preparation

The variability of density (S2 Fig), color (beige to red) and grain size (fine to coarse) of the clays used by Natufians to make figurines and cylinders (Figs 4-5) suggests that they were collected from a variety of sources. The exact characterisation is beyond the scope of this preliminary study. It will be later identified by petrographic peeling, a minimally invasive method applied to delicate Early Neolithic clay artefacts [e.g., 80]. Based on surface observations, the clay in the three discussed assemblages is predominantly dense, fine-grained and free of large inclusions, suggesting that the Natufians targeted specific naturally levigated clay deposits (e.g., slow-moving freshwater). Contrary to what is observed in the earliest baked pottery of the Levant (c. 8,500 cal. BP), at PPNB Kfar HaHoresh, where the local marl matrix was tempered with herbivore dung [81], our preliminary observations did not identify the inclusion of vegetal temper in the Natufian fabric. Rare natural inclusions such as small freshwater shells (Cf. Cochliopidae) (Fig 6F) suggest that clay was collected near freshwater sources. In addition, there are anthropogenic inclusions in the clay such as flint flakes (Fig 6 Bc), bone splinters (S8H; S10A,D; S16G-K; S17F Figs) and wood charcoal fragments (S10D,G,H; S11C-D; S16D Figs). These rare anthropogenic inclusions, undoubtedly unintentional, suggest that the manufacturers did not use the sediment directly available on the floors of their habitats (very rich in anthropogenic inclusions) but, rather, went to collect clay outside the settlements before preparing and modeling it in domestic areas. This pattern of acquisition, shared by all three communities, suggests that the Natufians sought out clays with particular plastic qualities, grain size and possibly also colors to suit this new mode of expression. Such acquisition practices are consistent with the context of the expansion of plastic mineral matters (PMM) technologies in Natufian architecture where clay and mud, sometimes deriving from outside the settlements, have been used in large quantities to coat or construct walls and floors of dwelling structures, domestic installations or storage structures, or even to accommodate the dead in plastered burial pits (see a synthesis in [82]). More importantly, this acquisition pattern is similar to that of Natufian clay ornaments modelled in the same sites [31].

**Fig 6:**
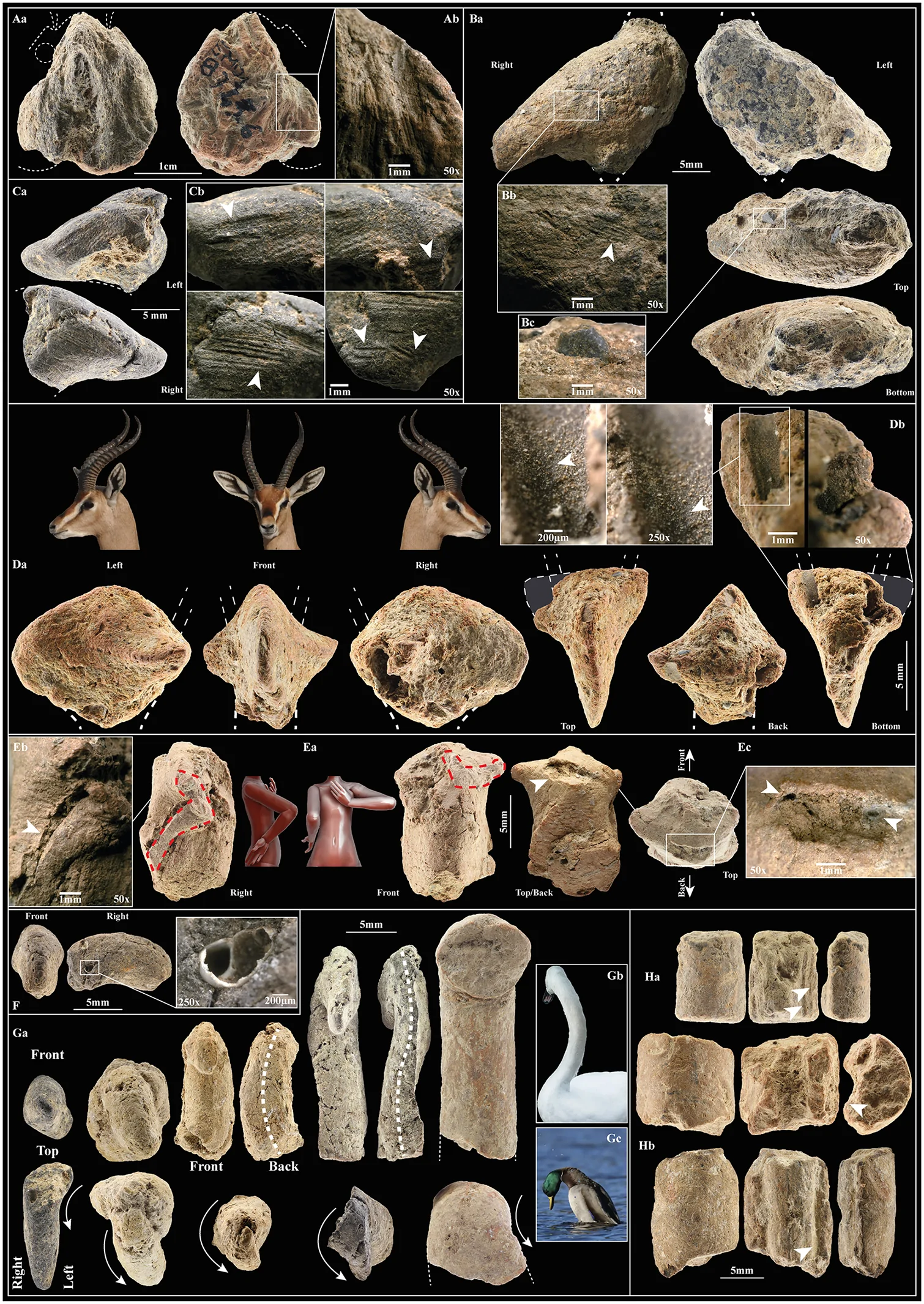
Shaping techniques of the clay figurines and cylinders. Final Natufian EM, A: The anthropomorphic figurine EM01 8745.6 of which the back is covered with plant straws imprints (50x). Ba: Ornithomorphic figurine EM97 6143 of which the left profile is irregular and not modelled; Bb: shaping marks left by a tool such as a vegetal stick (50x); Bc: flint flake embedded in the clay matrix (50x). C: Shaping marks on the zoomorphic figurine EM97 6140 (50x). Da: Zoomorphic figurine EM99 7121 at the back of which (Db) two insertions (c. 1 mm ⌀) might have hosted perishable materials to represent horns (suggested by dotted lines). Ea: Early Natufian anthropomorphic figurine EM75 4551.17 of which the arms have been modelled in a non-symmetrical position by shaping and incision (Eb); The head (Ec) is missing, and only the fracture outline where the neck was attached to the body is visible. Inside this fracture, in a symmetry on either side are visible two perforations (c. 500 µm ⌀) where perishable materials might have been inserted in the clay. F: Fragmented ornithomorphic clay figurine EM97 6104 with a small freshwater shell (Cf. Cochliopidae) embedded in the matrix (250x). Ga: Figurines from the Early to Final Natufian of EM (Fig 4F,H-K – seen from the front, top and back) with their heads modelled to be turned to the left (white curved arrows on top views) and two figurines with their cylinder neck vertically curved (dotted lines on back views) as can be the neck of waterfowls (Gb); Gc: Grunt-whistle courtship display performed by a male Mallard (*Anas platyrhynchos*). Fragments of cylinders where at least two distinct layers of cylindrical shaping can be seen in the Final Natufian of EM (Ha) and the Late Natufian of HT (Hb). (Photos and CAD L.D.).

### Heating of the clay

All the clay artefacts presented here have been heated in one way or another, otherwise, they would never have been conserved under the preserving conditions prevailing in the sites. In addition, it should be noted that nine of the fifteen figurines (Table 1) and 30% (n = 42) of the cylinders bear signs of contact with fire (blackened or reddened areas). Were those objects permanently hardened intentionally? The characterisation of the heat treatment of the Natufian clay figurations is beyond the scope of the present study. Future analyses, combining thermal demagnetization and Fourier-Transform Infrared Spectroscopy (FTIR) (see details of this combined approach to reconstruct firing history and estimate firing temperatures of clay artefacts in [83]), will allow the identification of accidental (i.e., post-depositional or destruction by fire), intentional (i.e., emergence of the ceramic technology) or passive baking (i.e., located in the vicinity of a heat source or left in the sun to dry). The emergence of ceramic technology during the Natufian seems possible given that such practices are attested in the Late Natufian by the baked (≤400°C) woman/goose figurine from Nahal Ein Gev II (Fig 1X) [46] and two baked (500–600°C) clay cylinders from Salibiya I (Fig 3E-F) [55]. Natufians commonly practiced pyrotechnology, sometimes in designated areas [e.g., 84], to produce lime plaster used as adhesive material or for funerary and domestic architecture (see a synthesis in [85], transform goethite into hematite [33], change the color and flaking properties of flint [86], as well as of bone pendants [87] and bone tools [88].

### Inventory

#### Ornithomorphic figurines.

The majority of figurines are ornithomorphic representations (11 of the 15 figurines; Fig 4E-O; Table 1; S8-S18 Figs; S8-S11 Movie) produced at both HT and EM from the Early to the Final Natufian. Among these 11 ornithomorphic clay figurines, we can distinguish at least two distinct modes of representation. The first, represented by two figurines from Early Natufian EM (Fig 4F,H; S4 Table; S8-S9 Figs), consists of modeling, without respecting natural anatomical proportions, a neck/body schematised in the form of a cylinder from which no appendages (i.e., wings, tail, legs) emerge. At the end of this cylinder, a schematic head is modelled to represent the profile of a bird’s head without transcribing clear anatomical details (i.e., eyes, beak separation). This head, formed by a more or less pinched curved cone, faces downwards at an identical angle on both figures (c. 36° angle). The only aspect that seems to reflect a naturalistic representation is the vertical curvature of the cylinder (Fig 6 Ga) reminiscent of the neck posture adopted by some waterfowl (Fig 6 Gb). A third cylinder discovered in the same context and broken at the level of the head, also curved downwards (Fig 4I; S4 Table; S11 Fig), could belong to the same figuration mode. The same attention to detail can be seen in marking the junction between the bottom of the head and the neck/cylindrical body, which is rather naturalistic, through one or more meticulous incisions in the wet clay using tools with a fine (from c. 200 µm to 250 µm wide), either sharp (S9A-D Fig) or rounded (S8C; S10B Figs) point. Looking at these three figures from above (Fig 6 Ga), we can also see that the heads have all been modelled to be turned to the left.

These schematic ornithomorphic figurines with cylindrical necks and bodies prefigure those carved in stone three millennia later, most of which depict birds of prey [79] in the PPNA villages of Mureybet (S25A Fig) [89] and Jerf el-Ahmar (S25B Fig) [90] in the Northern Levant, Nemrik 9 in northern Mesopotamia (S25F Fig) [91, 92], Hallan Çemi [93] and Körtik Tepe (S25E Fig) [94] in Anatolia. The concept of representing a head, whether stylised or naturalistic, at the end of a cylinder is a typical feature of Natufian iconography on bone and stone (Fig 1). In this respect, it seems possible that this mode of ornithomorphic representation is the extension, in clay, of a formal figurative convention shared on a cultural scale. However, there is a conceptual difference between clay and other materials. If, on most bone cylinder figurines (Fig 1B-D,F), the manufacturers felt it necessary to mark an artificial boundary between the cylinder and the figurative head with a transverse peripheral incision, this is not the case in clay figurines. It may suggest that the clay cylinders are probably not simply a support for the figures, but are an integral part of them, just like the head.

The second mode of ornithomorphic representation is observed on one figurine from Final Natufian EM (Figs 4E and 6B; S4 Table; S8 Movie). Here, unlike the figurines from the Early Natufian, the emphasis is on the naturalistic aspect of the representation, respecting natural anatomical proportions. The maker drew the profile of a bird, revealing a folded wing, a tail, a swelling in the breast area, and most probably legs and a head that are currently missing. Thus, in contrast to the majority of all Natufian figurations, which, although fragmented, suggests a preferential figuration of heads (Fig 1) (21), this figurine is one of the few to represent the entire body of the depicted subject. The maker meticulously represented the wing and tail feathers by marking the surface of the clay with long parallel incisions (c. 400 µm wide) with a different orientation between the wing and the tail (Fig 8B), as is the case in the living animal. The attention paid to the representation of feathers is reminiscent of the head of a bald ibis (*Geronticus eremita*) engraved in profile on a soft limestone plaque five millennia earlier, during the Geometric Kebaran of Ein Qashish South in the Jezreel Valley, the earliest depiction of a bird known in Southwest Asia (dated to c. 17,000–16,500 cal. BP) [5]. Leaving aside its unifacial mode of representation, the Natufian figurine anticipates a more schematic figure with a similar profile, carved on soft limestone a few centuries later, from the PPNA village Gilgal I in the Jordan Valley (S25D Fig) [58]. Of the same period, but on a monumental scale, there is a stylistic link with the birds of prey carved on the stone pillars of the ritual sites of Göbekli Tepe and Nevali Çori in Anatolia (see synthesis in [79]).

Seven fragments of ornithomorphic heads have been identified (Fig 4G,J-O; S4 Table; S11-S17 Figs). We can recognise in Late Natufian HT (Fig 4M-N; S10-11 Movie) and Early (Fig 4J; S9 Movie) to Final (Fig 4G,K,L,O) Natufian EM, echoes of the stylised profiles of cylinder figurines from the Early Natufian EM (Fig 4F,H) described above. The connection with the cylinder figurines can also be seen on the two head fragments where the neck is best preserved (Fig 4J,M). Here, as on the cylinders, attention is paid to marking the naturalistic junction between the lower part of the head and the neck, by one or more meticulous incisions in the wet clay, using tools with a rounded fine point (from c. 250 µm to 600 µm wide) (S11A; S17B-H-L Figs). Finally, on two fragments from respectively Early (Fig 4J) and Final (Fig 4K) Natufian EM the heads have been modelled, as on the cylinders, to be turned to the left (Fig 6.Ga) and, when the junction with the neck can be seen, the head has been turned downwards (c. 50° angle) (Fig 4J). The profiles of these fragments show that, while the beaks at HT appear to be exclusively pointed, at EM they are either pointed or rounded, while the similarity between the two sites can be observed in the two heads with a conical profile (Fig 4K-M). The one fragment from HT (Fig 4M; S4 Table; S17 Fig) which seems to be part of a figurine in the form of a neck/cylindrical body, attests to the spread, in space and time (Late Natufian), of this mode of representation.

There is, however, a variation at HT as unlike at EM, the bird’s head would have been oriented upwards (c. 117° angle) like the cylinder figurines of the same type from PPNA Nemrik 9 (S25F Fig) [91, 92] and Körtik Tepe (S25E Fig) [94]. The second ornithomorphic figurine fragment from HT (Fig 4N; S4 Table; S16 Fig) differs from the rest of the whole assemblage in several ways. Firstly, by the longitudinal extension of the neck, with no curvature like that of the figurines where this junction is preserved (Fig 4F,H-J,M), too rectilinear to resemble a profile figuration (Fig 4E), which suggests that the body of this bird was depicted in a different position, perhaps in flight, like the bone pendants from Early Natufian Wadi Hammeh 27 (Fig 1I) [25] and Fazael VI [95]. On the other hand, the figuration of this head is the only one to be naturalistic as regards its profile, its proportions and the meticulous symmetrical work of transverse incisions aimed at revealing the relief boundaries of a hooked beak, a head and a neck (S16A,J Fig). A group of parallel longitudinal lines (c. 400 µm wide) created by grooving after the clay had dried seems to have delimited the ovoid outline of an eye on the right profile (S16E Fig). On the left profile, the brown/black matter residue applied transversally might have figured an eye (S16B Fig). This asymmetrical treatment of zoomorphic anatomical details is reminiscent of what can be seen on some Pavlovian clay figurines [96].

Notably, ten of these eleven ornithomorphic figurines show traces of red colorant at various points on their surface (S4 Table). These colorants, most of which have not yet been characterised, ranging in hue from orange-red (S12B Fig) to bright red (S9C Fig), are preserved at different scales, from residue (Fig 7B-C) to widespread surface coverage (Fig 7D), which suggests that these figurines were initially partly or entirely colored. On the Early Natufian cylinder figurine from EM (Fig 4I; S4 Table; S10 Fig), the colorant is omnipresent on the outer surface (Fig 7 Ac) but absent in the internal section of the head fracture (Fig 7 Af). It was characterised by Scanning Electron Microscopy – Energy Dispersive Spectroscopy (SEM-EDS) as an engobe, a thin layer of orange-red clay rich in iron oxide (Fig 7 Ae) covering the surface of the figurine, whose light beige matrix is free of iron oxide (Fig 7 Ad). In detail, fine longitudinal and parallel marks (Fig 7 Aa-Ab) suggest that the wet engobe was applied to the surface of the figurine and smoothed longitudinally with a finger. The engobe has also enabled the maker to create a surface decoration in the form of a diagonal line free of colorant (c. 500 µm wide), running down towards the left profile, and located across the cylinder on the front of the figurine (Fig 7 Af-Ah). The absence of any tool marks at the bottom of this line and the morphology of its contours (S10C Fig) suggest that this decoration was created using a stencilling technique, probably with a plant fibre placed on the surface of the cylinder while the engobe was being applied. Although the technique of stencilling with engobe is currently a unique occurrence, this diagonal line decoration is similar to that printed or incised on numerous EWT, HT and EM clay cylinders throughout the Natufian culture (Fig 8E). To date, this Early Natufian figurine provides the earliest evidence of the existence of the engobe coloring technique.

**Fig 7:**
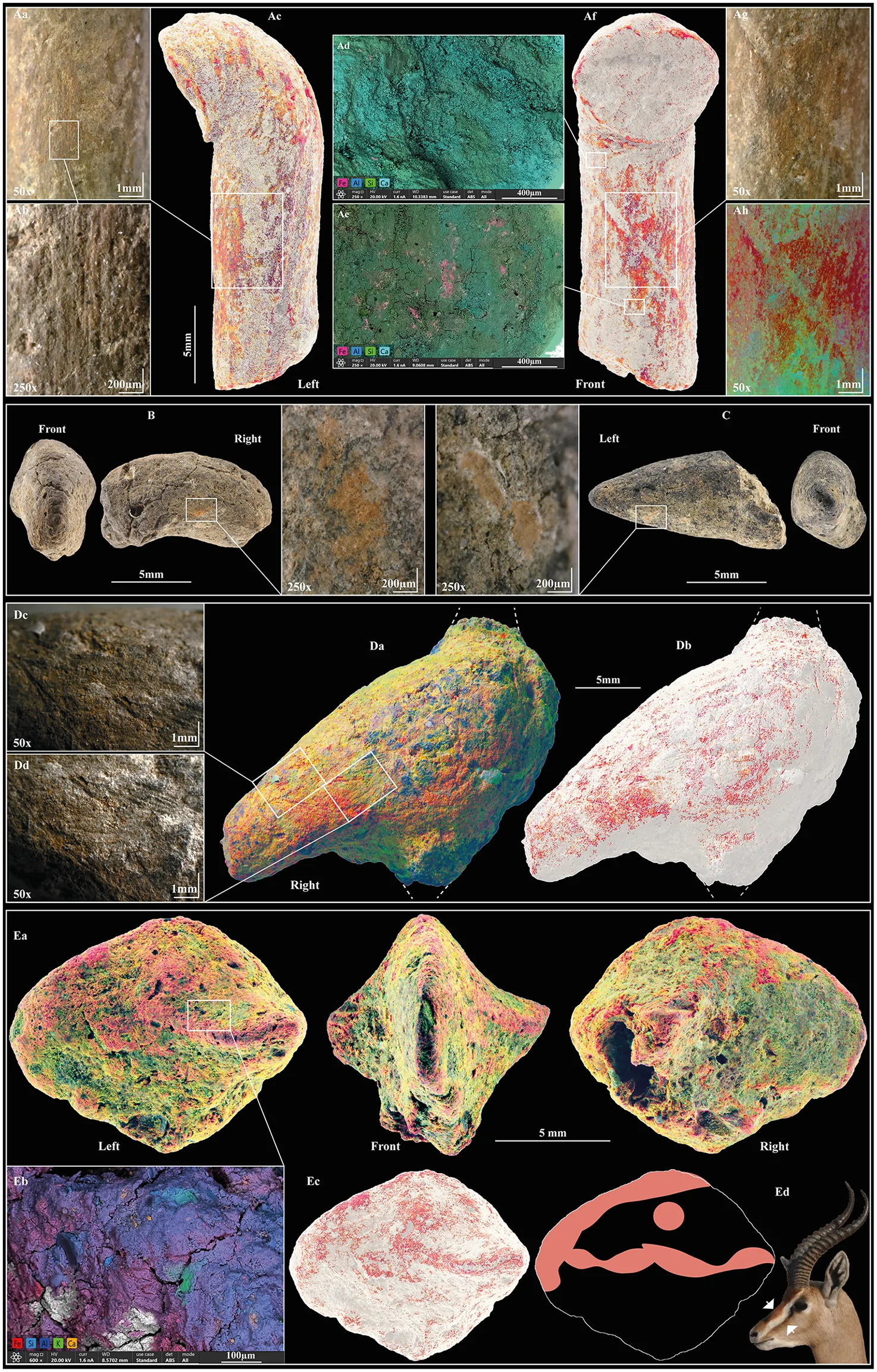
Surface coloration of the clay figurines and cylinders from EM. Fragmented clay cylinder figurine EM74 3399. Detail of the red residue application longitudinal marks (smoothing with fingers): Aa: 50x; Ab: 250x; Ac-Af: distribution of the red residues on the surface (from a Dstrech Lab view); SEM-EDS analysis (250x) suggesting Ad: the absence of iron where red residues are not visible; Ae: the presence of iron-rich clay (engobe) where red residues are visible; Ag: detail of the stencilled diagonal line decoration (50x); Ah: Dstrech (Lre) view of the same area. Detail (250x) of the reddish colorant residues on the surface of the ornithomorphic figurines fragments; B: Fragmented clay ornithomorphic figurine EM97 6104. C: Fragmented clay ornithomorphic figurine EM98 6600. Fragmented clay ornithomorphic figurine EM97 6143; Da: Dstrech (Lab) view exacerbating the presence of reddish residues on the surface; Db: Distribution of the reddish residues on the surface (from a Dstrech Lab view); Dc-d: Detail (50x) of reddish residue on the wing and the tale; Fragmented clay zoomorphic figurine EM99 7121; Ea: Dstrech (Lab) view exacerbating the presence of red residues on the surface; Eb: SEM-EDS analysis (600x) suggesting the presence of iron-rich clay (engobe) where red residues are visible; Ec: distribution of the red residues on the surface (from a Dstrech Lab view); Ed: possible original distribution of the red color on the figurine’s head compared to the fur color lines of a Mountain Gazella (*G. gazella*). (Photos and CAD L.D.).

**Fig 8:**
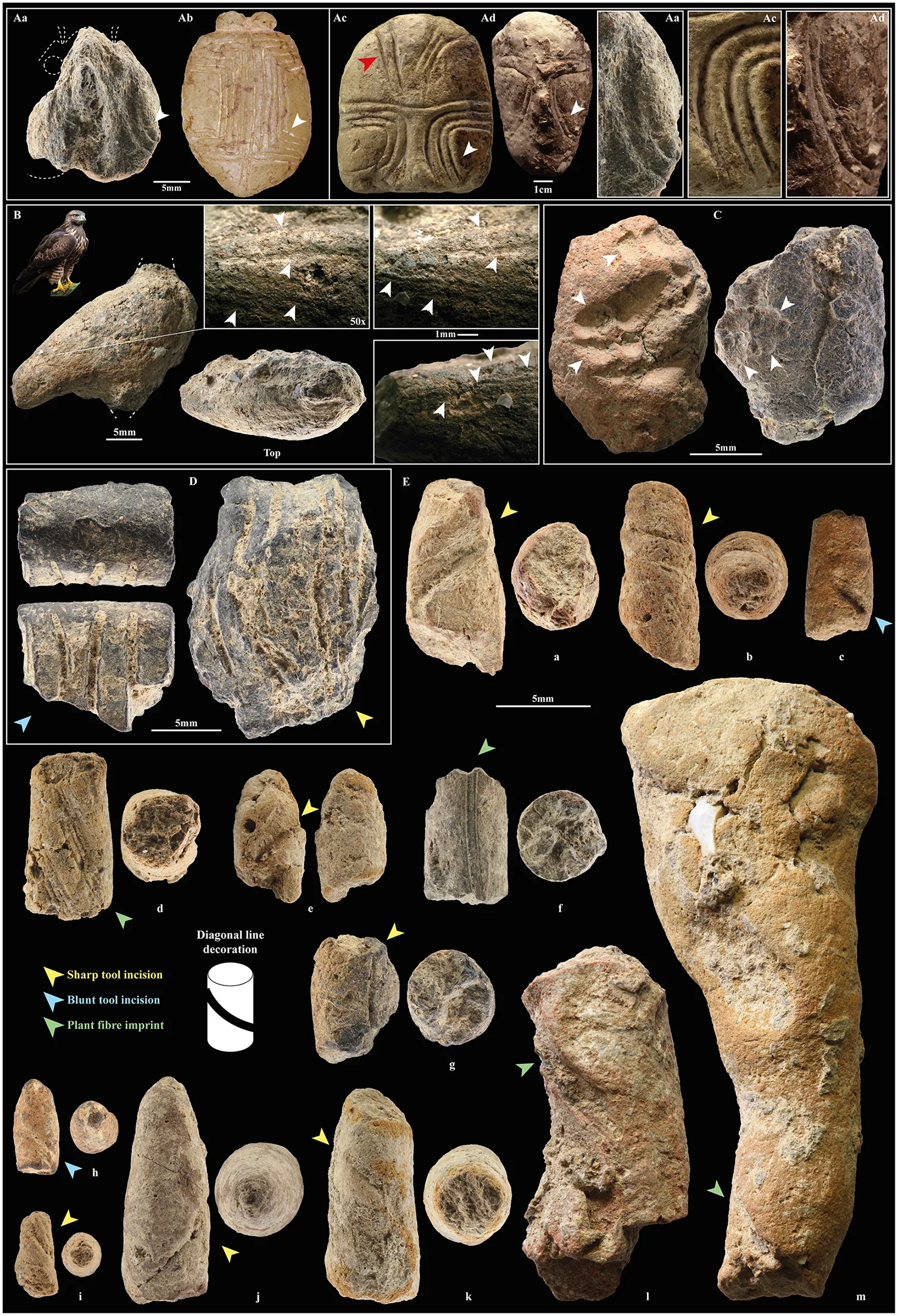
Relief decorations of the clay figurines and cylinders. EM, on the faces of the anthropomorphic figurines, the similarity of the parallel curved lines pattern shaped on Aa: clay figurine EM01 8745.6; and incised on limestone figurines; Ab: EM75 4101; Ac: EM59 1266 (upper decoration indicated by the red arrow); Ad: EM61 1777; B: Fragmented clay ornithomorphic figurine EM97 6143, decoration of parallel longitudinal lines printed in the wing (indicated by arrows) with a blunt tool and suggesting feather representation (250x); C: Impressions made on clay artefacts from Late Natufian HT (HT87 2243) and Final Natufian EM (EM01 8478) by using the articular ends of birds or small mammals bones; D: Clay artefacts from Late Natufian HT decorated with parallel lines incised with a blunt tool (HT87 2193) and a sharp tool (HT88 2385); **E**: Diagonal line decoration incised with sharp and blunt tools or imprinted with a vegetal fibre on clay cylinders from EWT, HT and EM. (Photos L.D. and The Israel Museum).

To conclude, birds were modelled throughout the Natufian culture, at least at HT and EM. Although birds during the Natufian represented a significant proportion of the hunted game [e.g., 97], appeared in funerary deposits [35], were used as raw material for feather ornaments [46], bone tools and ornaments [28,98], or sound instruments [34], they had been under-represented in Natufian iconography. Only three bone pendants from Early Natufian Wadi Hammeh 27 (Fig 1I) [25] and Fazael VI [95] are known, carved to represent a flat, two-dimensional profile of a headless bird in flight with the feathering effect of a tail and wings. It was thought that this iconography only appeared in Southwest Asia from the Early Neolithic onwards. Indeed, during the PPNA, birds became a major subject of zoomorphic representations on stone in the form of figurines (S25 Fig) [58,91–93], statues, engraved objects [e.g., 90–94, 99] and bas-reliefs on monumental pillars [e.g., 99] (see synthesis in [79]. The discovery of these 11 Natufian ornithomorphic figurines, together with the woman/goose figurine from Nahal Ein Gev II (Fig 1X) [46], anchors the emergence of bird iconography in pre-Neolithic cultures. Furthermore, when it emerged, bird iconography seemed closely linked to clay, which could suggest a specific link between the material used and the subject depicted.

The stylised mode of representation shows continuity, stylistic tradition, expressed in the body/cylindrical neck. Previously, such a chrono-stylistic continuity was observed in zoomorphic figurations on functional objects (artiodactyl bone sickle hafts and hoof-shaped stone pestles) [23,25,38–41]. In terms of the subjects depicted, this tradition is part of the Natufian zoomorphic style of expression, which consists of representing animals in an elusive and ambiguous way [1,25,38,40] which prevents the uninitiated observer from recognising a specific bird species. However, from the shape of the profile of the heads and beaks two groups of birds can be distinguished (S4 Table). One group (n = 6) with curved heads on long necks and rounded beaks, only represented at EM in the Early and Final Natufian, probably of the Anatidae family (i.e., ducks, geese and swans), which are the birds most often hunted in this settlement [97,100]. The second group, with a straight head and pointed beak, is represented by two figures from the Late Natufian HT (Fig 4M) and the Final Natufian EM (Fig 4K). This naturalistic mode of figuration seems to represent other bird species. The hooked beak of the HT figure (Fig 4N; S16 Fig) is, following the assignment of similar PPNA figurines [e.g., 79], a characteristic associated with figurations of birds of the family Accipitridae (i.e., small to large birds of prey) hunted by the inhabitants of this site [101]. The same applies to the EM figurine (Figs 4E and 6B; S8 Movie), whose profile and anatomical proportions suggest the representation of a falconiform (Fig 8B), birds hunted on the site for the use of their talons in adornment, and whose calls have been imitated by bone aerophones [34,97]. This coexistence of representations of waterfowl and bird of prey seems to prefigure what would later be observed in the PPNA (see synthesis in [79]).

In EM, besides the stylised mode of expression shared on an inter-community level, the makers transcribed, from the Early to the Final Natufian, a local figurative formal convention which consisted of modeling the heads of the birds tilted downwards and turned to the left (Fig 6 Ga). This posture detail is important because most of the Natufian figurations were considered to lack any inherent scene or narrative [25,50]. This absence of an explicit discourse led François Valla [48] to consider Natufian figurines as “*mythogrammes*”, more akin to ideography than pictography [102], that could only be understood with the aid of a verbal accompaniment, which is lost to us. However, unlike European figurative expression, Natufian zoomorphic figurations have never been approached from the angle of the ethology of the animals they represent. Yet thanks to this approach, developed by André Leroi-Gourhan [103], we can observe that the heads of artiodactyls figured on bone from Early Natufian Kebara (Fig 1C,E,F) or el-Wad (Fig 1G) are ostensibly oriented upwards and some have their ears laying back. This upward posture is similar to the courtship display adopted by the males of many artiodactyl species, including the mountain gazelle (*Gazella gazella*) [104] Natufians’ favourite prey [e.g., 105], during the breeding season (i.e., autumn in the Southern Levant). In the same way, a limestone zoomorphic figurine from Early Natufian Wadi Hammeh 27 (Fig 1J) could depict tongue flicking from slightly opened mouth that occurs in courting males of many ungulate species [104]. Obviously, the ethological approach can bring Natufian figurations out of their apparent muteness and reinterpret them as pictograms (i.e., narrative figurations) by identifying the behaviour of the animals depicted (e.g., the mating posture of the gander on the clay figurine from Nahal Ein Gev II [46]). It also sheds light on the manufacturers’ will to depict a snapshot of animal behaviour to trigger in the observer’s imagination the circumstances that cause it, one of the figurative techniques used by animist imagery to convey animal subjectivity [106].

Birds’ non-verbal communication is mediated by a limited repertoire of displays, with formally restricted angles of head orientation playing an essential role [107]. In the case of Anatinae (i.e., surface-feeding ducks) hunted preferentially by the EM Natufians [e.g., 97] and whose bones were used to make aerophones [34], the association of motor pattern (i.e., sequence of movements) bringing together the lowering and transverse movements of the head corresponds to the grunt-whistle courtship display practised by males during pair formation behaviour (Fig 6.Gc) [108]. It is practised by many species of Anatinae [109] at the first stage of the breeding season (i.e., end of winter/spring in the Southern Levant). At that time, any unpaired females attract the attention of males. A flock of suitors surrounds the female, parading courtship displays in which they extend their body vertically and lower their heads so that the beak scoops the water surface and, in a transverse movement, throw up a shower of droplets in a wide arc (Fig 6 Gc) always aimed at the targeted female [108, 109]. Accordingly, we suggest that by modeling Anatidae with the neck/body frozen in a vertical position and the head lowered in a transverse movement, the EM Natufians wanted to portray a trademark of the courtship display of surface feeding ducks. The depiction of this ‘suspended motion’ [106] suggests that, despite the small size of the figurines (Table 1), the birds are depicted in full maturity as male individuals in a reproductive context, portraying a specific calendar period, the end of winter/spring breeding season. Cross-cultural ethnographic records have shown that such association of animal behaviour in a specific context is the methodological basis of non-literate societies’ animal identification. In the resulting knowledge, stored and transmitted in ritualised performance (e.g., songs), storytelling about birds dominates all the other animal groups [110]. For example, due to meticulous observations and carefully accumulated knowledge on their courtship displays, ducks are role models for many Native American mating rituals [111]. Accordingly, the ornithomorphic figurative formal convention of EM can be interpreted as evidence of the Natufians’ ethno-ornithological knowledge.

#### Artiodactyl figurines.

Two clay fragments of zoomorphic figurines from the Final Natufian of EM (Fig 4A-B) seem to portray artiodactyls, symbols usually carved on stone and bone. The first is a schematic head with a triangular profile and a relatively smooth surface lacking any specific anatomical details (Fig 4A; Table 1; S4 Fig; S1-S2 Movie). It is broken at the neck and at its proximal part and was modelled in a fine clay exhibiting, over its entire surface, blackened areas which indicate contact with fire. All the facets were evenly modelled using a tool similar to a vegetal stick, judging by the succession of parallel line impressions (Fig 6 Cb), and with a symmetry fitting natural anatomical proportions. The preserved part does not allow us to identify a particular posture. The better-preserved right profile (S4 Fig), reveals the naturalist extension (i.e., curved) of a neck but it is not clear whether there was also a body. Residues of red colorant, not yet characterised, visible at several points on the surface of the head (S4 Fig), suggest that the figurine was colored red. With its pointed profile and schematic appearance, this head fragment is reminiscent of the artiodactyl-like figurations on bone from Late Natufian Nahal Oren (Fig 1 Ab,D).

The second fragment has been modelled in a fine to coarse light brown clay with no fire traces on its surface. It is an almost complete head, broken at the neck, with a generally circular cross-section (Fig 4B; Table 1; S5 Fig; S3-S4 Movie). Here too, the style is schematic, and the surface lacks facial features. However, this figurine is shaped differently from the previous one: the maker modelled the head to resemble a lozenge, with the rounded muzzle and occipital protruding horizontally and the rounded forehead and neck vertically. This lozenge profile is replicated from the front by modeling, using blunt tool incisions (U section c. 350 µm wide) (S5F Fig), ears held horizontally sideways. This ear posture, associated with the threat-dominance displays (i.e., indicating alert, readiness for fighting) in artiodactyls [104], is unique among all Natufian zoomorphic representations in that the ears, when they are depicted, are always either raised upwards (Fig 1D,G), hanging down (Fig 1E) or laying back (Fig 1B,C,F,H). Moreover, when viewed from the front, this posture is accentuated by the exaggeratedly pinched muzzle, digressing from the natural anatomical proportions. Behind the ears are two symmetrical insertion impressions (Fig 6 Da), both oriented horizontally along an axis parallel to the profiles of the head (c. 17° angle to the horizontal axis from the muzzle to the occipital bone) and vertically, upwards towards the back of the head (c. 115° angle to the horizontal axis from the muzzle to the occipital bone). Extending them, these insertions take the form of a ‘V’ above the head when the figure is viewed from the front (Fig 6 Da). The better-preserved insertion impression, on the left profile (Fig 6 Db), is a cylindrical impression (c. 1.2 mm wide) on the surface of which we can see, microscopically, fine longitudinal and parallel lines imprinted by the element inserted in the wet clay. It therefore seems reasonable to suggest that these two insertion impressions correspond to two elements made of perishable materials (e.g., vegetal twigs) inserted into the clay to represent the horns/antlers of the animal depicted. It should be noted that the placement of these appendages behind the ears is in keeping with the schematic style of the figure, as they do not correspond to the actual positions of the horns/antlers of the artiodactyls, which are higher up on the skull.

In the current state of research, this figurine is unique being the only Natufian figurine with depicted horns/antlers, and, together with the woman/goose figurine from Nahal Ein Gev II (Fig 1X) [46], it is the earliest composite figurine (i.e., combining several materials) in Southwest Asia. This technique was later used in Neolithic Çatalhöyük to depict the removable antlers of a deer clay figurine [112]. In addition, the head is covered in red colorant (Fig 7 Ea) characterised by SEM-EDS as an engobe, i.e., a thin layer of clay rich in iron oxide (Fig 7 Eb) covering the figurine surface (S5A Fig) whose matrix is free of iron oxide. On the better-preserved left profile (Fig 7 Ec), there are areas of concentrated engobe, suggesting that it was not applied randomly. In fact, like fur color lines, the engobe is concentrated in two lines, one at the top of the head and the other lower down, on a horizontal axis between the muzzle and the ear, in the middle of which a circular concentration could represent an eye in an anatomical position (Fig 7 Ed). As a result, the profile of the head, the arrangement of the horns/antlers (Fig 6 Da) and the fur decoration formed by the engobe (Fig 7.Ed) suggest that the animal depicted could be a mountain gazelle (*Gazella gazella*). Such depiction would be a first because although this artiodactyl was the favourite prey of the Natufians [e.g., 106] and it played an important role in their techno-symbolic sphere, as attested by funerary deposits of horns [35,48] and the ornaments made from its bones [28–30], the elusive and ambiguous aspect of the Natufian zoomorphic expression had not made it possible to identify the figuration of a gazelle [25]. Together with the contemporaneous naturalistic Accipitridae figurine from EM (Figs 4E and 6B; S8 Movie) and the woman/goose figurine from Nahal Ein Gev II (Fig 1X) [46], this raises the question of whether these figurines might be evidence of a trend, at the end of the Natufian, to overcome the ambiguity of zoomorphic expression by using new figurative techniques (e.g., mixed materials and engobe decoration).

#### Anthropomorphic figurines.

Two anthropomorphic figurines from EM (Fig 4C-D) attest that these symbols usually carved on bones and stones (Fig 1 Aa,O-W) had been depicted also on clay. The first figurine, dated to the Early Natufian (Fig 4C; Table 1; S6 Fig; S5-S6 Movie), had been modelled on a fine, light brown clay with no traces of contact with fire or colorant residues. It depicts the upper half of a body, consisting of a sub-cylindrical trunk at the top of which shoulders and two arms have been modelled in a schematic style. It is impossible to assess whether the individual is represented in a seated or standing position. The back is relatively flat, with traces of transverse smoothing (Fig 6 Eb), while the abdomen is ostensibly convex, in contrast to all the Natufian carved anthropomorphic busts known today. An ovoid fracture surface (c. 4.7 x 2.2 mm) in the middle of the shoulders and at the top of the back (Fig 6 Ec; S6 Movie) suggests that the figurine comprised also a neck and head. Two insertion impressions in clay (c. 500 µm wide), located symmetrically at the ends of the fractured neck, suggest that the junction of the neck and trunk perhaps was ‘reinforced’ with vegetal fibres, a technique seen in numerous later contexts [113]. While this trunk/shoulders/head configuration is classic in Natufian anthropomorphic expression, known from several figurines [25] including one from EM (Fig 1R) [44], the representation of the upper limbs is exceptional, as until now only two other figurines with limbs were known (Fig 1V,X). In the EM figurine, the posture and modeling of the upper limbs are asymmetrical (Fig 6 Ea; S6 Fig). The left side is partially blunted, but the arm can still be seen abducted horizontally, with the elbow distanced from the body (Fig 6 Ec). The forearm is adducted and brought back to the chest, where an angle in the modeling suggests that the hand is pointing towards the head. The left upper limb is modelled mainly in rounded volumes with the contours of the forearm emerging in bas-relief, but there are also incisions to separate the elbow from the body, made using a rounded tool (U section c. 350 µm wide) (S6A Fig). The right upper limb is held at the side of the body in what appears to be a constrained position. The arm is flexed while the forearm is extended, and the hand, figured in a pointed form, just the contour without detailing fingers (S6G Fig), is by default pronated. This limb was modelled in rounded bas-relief, the contours of which were cut out of the wet clay using a pointed tool (Fig 6 Eb). There are also incisions to shape the angulation of the elbow (S6Fb Fig), made using a rounded tool of which morphology is similar to that used on the left elbow (S6A Fig). This asymmetrical posture, combining the left forearm pulled in toward the front of the body and the right arm held along the side of the body, is similar to that of the woman/goose figurine from Nahal Ein Gev II (Fig 1X) [46]. In addition to the volumetric modeling, two distinct surface treatments focus on different parts of the figure’s body. The right side of the abdomen is marked with at least nine vertical and parallel shallow incisions (U section c. 500 µm wide) under the right forearm (S6H Fig) made by a blunt tool. Such a pattern, with no overlap between the lines imprinted in the wet clay, suggests the use of a comb-like object (i.e., the distal end of a small bird tarsometatarsus [114]) in a stab-and-drag gesture. This pattern recalls the twelve vertical and parallel lines incised on an anthropomorphic limestone figurine from PPNA Gilgal I, interpreted as representing a garment [58]. The second surface treatment is located on the chest, between the left forearm and the right arm (S6D-E Fig). Here, two shallow impressions were made in the wet clay (c. 2.3 mm wide) in the same orientation, and each took the form of two symmetrical ovoid depressions between which a line emerges. This characteristic shape is reminiscent of the articular condyles of bones. It suggests that these impressions were made by using the articular end of a bird or a small mammal dry bone (e.g., the distal end of a bird pedal phalange). Such stamped impressions, using different bones, were also made on clay objects from Late Natufian HT and Final Natufian EM (Fig 8C), which suggests that this technique was continuous over time and space. The impressions on the figurine appear too shallow and barely visible to the naked eye to be considered a decorative motif. We suggest that the repeated gesture of delicately and precisely touching the figurine with a small dry animal bone was not related to an intention to shape it (i.e., a non-technical interaction between the manufacturer and the figurine). Such practices are known from the PPNB to the Pottery Neolithic (PN) of the Levant, where many clay representations of aurochs incorporate embedded flint blades or flint punctures in vital body parts [e.g., 115,116].

The second anthropomorphic figurine from EM, dated to the Final Natufian (Fig 4D; Table 1; S7 Fig; S7 Movie), has been modelled in a fine, light brown clay with blackened or reddened areas on the surface, indicating contact with fire. Residues of red colorant, yet unidentified, visible at several points on the surface (S7E Fig) suggest that the figurine was colored red. It features a stylised anthropomorphic face the right side of which is partially broken. The appearance of the proximal and distal sections suggests that they form the original limits of the artefact, indicating that the face probably functioned independently without being connected to a body, as is the case with many anthropomorphic Natufian figurations made of bone (Fig 1 Aa) or stone (Fig 1S-U). The profile of the figurine is plano-convex, with only the face (Fig 6 Aa) and its contours (S7A Fig) modelled. The side opposite to the face is covered with plant fibre imprints (not yet characterised), more or less organised and oriented in a parallel manner (Fig 6 Ab), possibly originating from the surface (i.e., mat) on which the wet clay was placed when the maker modelled the face. If reconstructed symmetrically (Fig 4D), the original contour of the face is ovoid overall, with two lateral notches at the top and a central notch at the bottom. The face is symmetrically organised around a central vertical axis formed by a protruding nose, disproportionately large to the rest of the face. The proximal part of the nose has become blunt, making it difficult to reconstruct its original shape and relationship with the central notch that forms the base of the face. By comparison, based on the only Natufian fully represented face on a calcite figurine from Early Natufian EWT (Fig 1P), the mouth has been incised directly under the nose and over a width identical to it. Thus, the notch under the nose of the clay figurine could correspond to the demarcation of a mouth, implying that only the upper part of the face has been depicted. This configuration would explain the absence of a chin (Fig 1 Aa,P) or at least the proximal pointed part of the face (Fig 1S,U). On the left side of the face, which is better preserved, an eye can be seen at the top, modelled as a semi-spherical protuberance. Fine longitudinal and parallel traces suggest that the eye was modelled with a flat and fine vegetal(?) tool (c. 200 µm wide) (S7C Fig). The representation of eyes is scarce on Natufian anthropomorphic figurations (Fig 1 Aa,P,X), and this mode of representation in semi-spherical volume is more reminiscent of eyes in the zoomorphic representations (Fig 1 Ab,C,D,G). It is worth noting the striking resemblance in the organisation of the face, with the anthropomorphic head carved on a bone handle from the Late Natufian at Nahal Oren (Fig 1 Aa) with a disproportionately large nose and small, semi-spherical eyes. Under the eye modelled in the clay, there is a modelled decoration comprising three open concentric and parallel vertical incisions, made with a blunt tool that opens toward the lateral margins. These incisions create two rounded bulges in their centre (Fig 8 Aa; S7F Fig). Despite the fragmentary state of the right side, a visible incision shows that this decoration was modelled symmetrically, suggesting that there was also a right eye.

This arched pattern is characteristic of Natufian non-figurative expressions on ground stone tools (e.g., shaft straighteners from Nahal Oren [38]; vessels and mortars from EM [43]) or architectural elements (e.g., the incised stone slabs of ritual building 2 in Early Natufian Wadi Hammeh 27 [117]) and interpreted as a representation of the natural geometric decoration of the carapace of the Spur-thighed tortoise (*Testudo graeca*) ([117], see a synthesis in [25]) whose importance in the symbolic sphere is attested by deposits in funerary contexts [35,48]. The only zoomorphic figurine previously known in EM (Figs 1N and 8.Ab), carved on calcite and dated to the Early Natufian was interpreted either as a tortoise ([118] or an insect [19]) and has the same decoration meticulously incised to the same scale, as the clay figurine, in symmetry along a central vertical axis. The arched pattern can be found on two larger anthropomorphic limestone figurines, which represent stylised faces in the Early (Fig 8 Ad) and Final (Fig 8 Ac) Natufian of EM [43, 44]. Here, the decor is arranged symmetrically to form a central T-shape, which represents the intersection of the eyebrow ridge horizontally and the nose vertically, a classical synthesis of facial anatomy in Natufian anthropomorphic expression (Fig 1U) [45].

Despite stylistic and morphometric differences, there are significant similarities between the clay figurine (Fig 8 Aa) and the contemporary limestone figurine (Fig 8 Ac). Firstly, by choosing to depict only the upper part of the face, above the mouth, and secondly by decorating the upper part of the head. This upper decoration can be seen on the limestone figurine above the horizontal eyebrow ridge. It is distinct from the incised eyebrow ridge because it does not intersect and is formed by two groups of parallel incisions arranged symmetrically in a V-shape (Fig 8 Ac). The upper decoration, less precise in its execution and symmetry than the lower T-shape from which it is disconnected, does not seem to represent the anatomy of the face. Its upper implantation position is similar to that of the two notches on the top of the head of the clay figurine (Fig 8 A1). There, the use-wear on the outlines of the preserved left notch (S7B,H,I Fig) confirms that it was part of the original modeling with a vertical insertion impression of a conical profile (outer diameter c. 1,6 mm) (S7H Fig). The walls of the insertion show spiral marks, which, together with its conical profile, suggest that it was modelled by rotating pressure on the wet clay and not by inserting an object that would have remained fixed to the figurine, as is the case with the contemporaneous gazelle head (Fig 6 Db). Because of its vertical orientation and the absence of an anchoring point, it does not appear that this layout worked with a suspension system for using the figurine as a pendant. Accordingly, we suggest that this receptacle was modelled to affix removable headdress decorations made of perishable materials (i.e., grasses, string, hairs or feathers). In case this decorative notch was modelled in symmetry also on the missing right side, perhaps on the stone figurine as well, the double organic headdress decoration would have been represented by the upper incisions (Fig 8 Ac). These practices are in line with what was later observed in the PPNB Tell Seker al-Aheimar [119] and Çatalhöyük [120], where the heads of anthropomorphic clay figurines were marked with symmetrically arranged lateral insertions to affix removable headdress decorations made of perishable materials. We can therefore suggest that the EM clay figurine is a reproduction, in a more naturalistic style and a perishable material headdress decoration, of a stylistically distinctive and contemporaneous face variant engraved in stone (Fig 8 Ac). We can trace the origins of this stylistically distinctive face variant*,* specific to the inhabitants of EM, at least to the end of the Early Natufian (Fig 8 Ad) and perhaps even earlier, if we consider that the calcite figurine from the beginning of the Early Natufian (Fig 8 Ab), could be a stylised anthropomorphic face whose headdress decoration would have been depicted in the form of two globular appendages. As a result, the reproduction of this stylistically distinctive face variant in EM for thousands of years across many generations allows us to characterise it as an iconic sign depicting a historical or imaginary character who must be recognised as such, at least by initiated observers, due to figurative conventions [106]. The figurative method of iconicity would thus have enabled the inhabitants of EM to identify the character whose presence was being established in the artefact and to know its role in the oral tradition, an essential condition for effective mythology and cosmogony [106].

#### Cylinders.

We identified 139 fragments of modelled clay artefacts (EWT n = 39; HT n = 43; EM n = 57) classified to the general category of cylinders (Fig 5; S3 and S5 Tables; S18-S24 Figs). These are exclusively section fragments, of which 47.9% (n = 59) have at least one original end preserved (S3C Fig): round (n = 20) (Fig 5 Bk) or pointed (n = 39) (Fig 5 Ah). Broken sections are oblique (63.9%) (Fig 5 Ak) or transversal (36.1%) (Fig 5 Al) in profiles, which could result from intentional or unintentional fracturing processes (S3B Fig). Traces of contact with fire in the form of blackened or reddened areas are observed on 30% of the fragments. The overall cylindrical profile of the artefacts gives the assemblage coherence as a category despite the metric variability of these small elements (Length x̄ = 12.11 ± 5.4 mm; Width x̄ = 5.85 ± 2.69 mm; Depth x̄ = 5.11 ± 2.55 mm; Weight x̄ = 0.55 ± 0.78g) (S5 Table; S2 Fig). On the other hand, the homogeneity (t-test p > 0.01) of the cylinders density from all three sites (x̄ = 1.19 ± 0.37g/cm^3^) (S5 Table; S2 Fig) suggests that there is no fundamental difference between the clays used or the way of modification. On a local scale, while the HT and EM fragments have similar dimensions (t-test p > 0.01), those from EWT are distinguished by being larger (t-test p < 0.01). This difference in EWT could either result from unintentional phenomena (e.g., different post-depositional fragmentation processes) or reflect a different scale of figuration in that site. In terms of morphology, the fragments were modelled in the shape of the cone (45.8%) (Fig 5 Ah) and the cylinder (54.2%) (Fig 5 Aj), more or less, curved or flattened (S3A Fig), in similar proportions in all three sites. In addition to this communality of forms, there is also a communality of modeling techniques. Indeed, the cylinders were either modelled from a single hand-rolled clay core or, in an additive process, a core was extended and enlarged by the addition of a second (Fig 6 Hb) and even a third layer of clay (Fig 6 Ha). This technique will be used millennia later for modeling the different body parts of Yarmukian clay figurines (c. 8,400–7,800 cal. BP) [69]. Interesting to note, also in Upper Palaeolithic Europe, the Pavlovian and Epigravettian clay figurines were modelled in an additive process [73]. Most surfaces are very even and appear to have been delicately smoothed. In the three sites, all along the duration of the Natufian, for at least 23 cylinders (16.5%) (Fig 8E), modeling is accompanied by surface decoration in the wet clay in the form of one or more diagonal or vertical lines incised with a sharp (Fig 8 Ea,b,e,g,i,j,k) or blunt tool (Fig 8 Ec,h) or by imprinting the piece with a plant fibre (Fig 8 Ed,f,l,m). This decoration is reminiscent of the diagonal slip stencil on the possible ornithomorphic figurine from Early Natufian EM (Fig 7A) and the incised and printed decorations on two fragments of a clay cylinder from PPNA Gilgal I [58]. They are also reminiscent of many cylindrical or tronconical stone anthropomorphic figurines whose waist is marked by a transverse incision in the Natufian of EM (Fig 1R) [44] and later in the PPNA, where these ‘belts’ are interpreted as a visual marker dividing these schematic bodies [121]. Other marks on a cylinder from Final Natufian EM (Fig 5.Ai - EM04 9631) resemble a longitudinal series of fingernail prints. This decoration was organised similarly on a clay cylindrical anthropomorphic figurine from PPNA Gilgal I [58]. There are also at least five cylinders of EWT, HT (Fig 8 Ee) and EM (Fig 5 Al) on the surface of which one can observe transverse insertion impressions in the clay, which suggests that, as on the figurines (Fig 6D; S7H Fig), composite decorative elements made of perishable material may have been attached to these objects. In addition to these decorations, at least 31 cylinders (22.3%) showed traces of red colorant (e.g., Fig 5 Bj), not yet characterised, visible at several points on their surfaces and suggesting that cylinders were partially or totally colored red. The longitudinal marks visible on some cylinders (Fig 9 Ha) suggest that this coloring, maybe in the form of engobe as on the figurines (Fig 7 Ab), was applied by finger smoothing. Thus, in all three sites, the similarity of aesthetic canons, modeling and decorative techniques suggests the existence of a figurative convention shared by their Natufian manufacturers.

**Fig 9:**
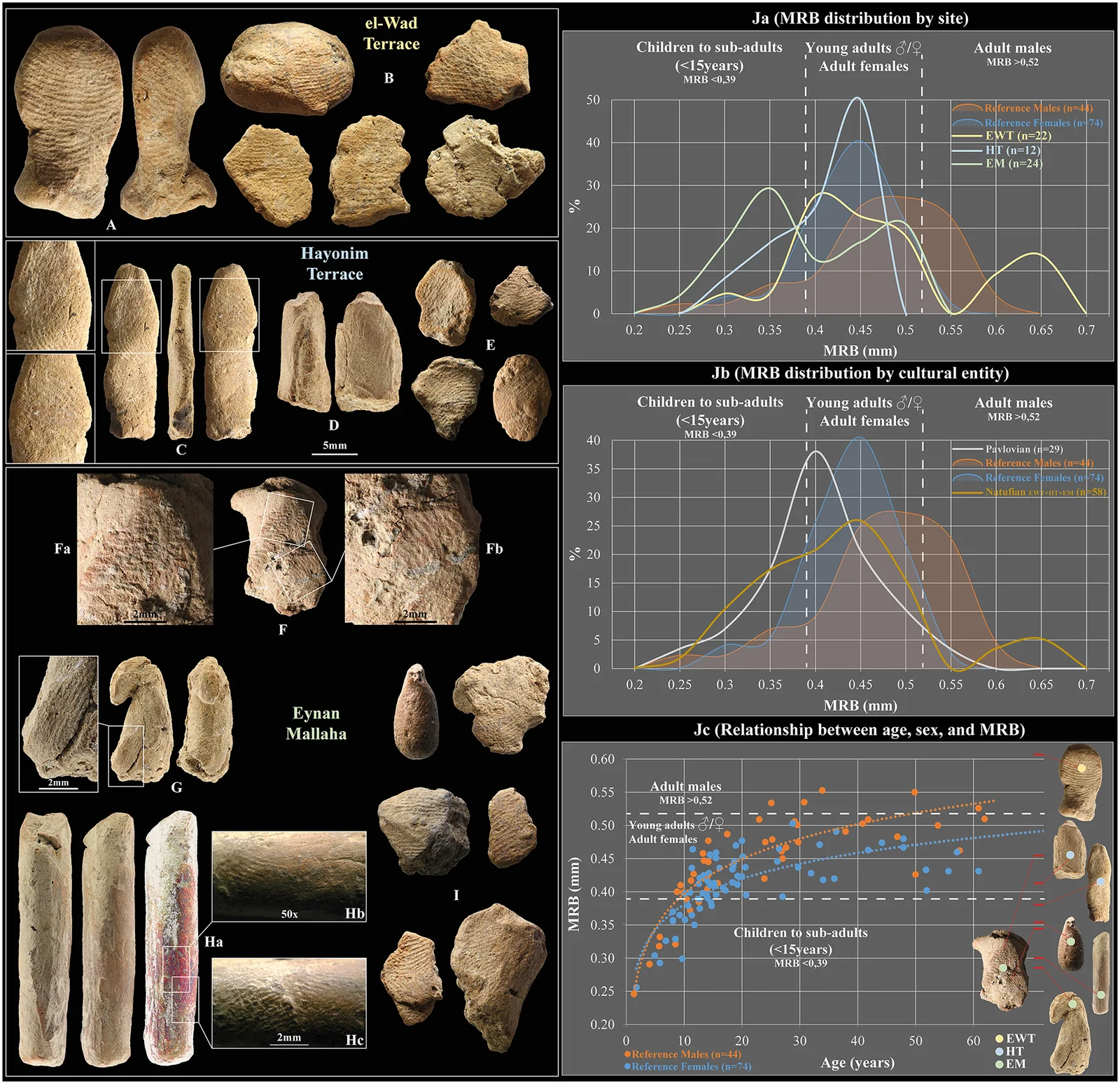
Sample of the fingerprints identified on the clay figurines, cylinders and shapings. EWT, Early and Late Natufian, A: cylinder EWT07 Q8d Bsk.51 with the fingerprints of an adult male; B: Shapings with the fingerprints of children to adult males. HT, Late Natufian, cylinders HT81 546 (C) and HT80 336 (D) with the fingerprints of sub-adults to young adults (♂or♀)/adult females; E: Shapings with the fingerprints of children to young adults (♂or♀)/adult females. EM, Early Natufian, F: anthropomorphic figurine EM75 4551.17 with (Fa) the fingerprint of a young adult (♂or♀)/adult female and (Fb) the fingerprint of a child/sub-adult (♂or♀); G: ornithomorphic figurine EM75 4585 with the fingerprint of a child; Ha: cylinder EM72 2261 covered by a red residue (exacerbated by a Dstrech Lab view) smoothed by the finger of a child; I: Final Natufian cylinder and shapings with the fingerprints of children to young adults (♂or♀)/adult females. J: Mean Ridge Breadth (MRB) measured on Natufian fingerprints compared to the reference experimental dataset of Králík and Novotny [122], the dotted lines mark the exclusive limits of the different demographic groups in the reference dataset; (Ja) Comparison of the MRB distribution between EWT, HT and EM; (Jb) Comparison of the MRB distribution between the Natufian fingerprints and the ones measured on 29 Pavlovian clay objects [123]; (Jc) Relationship between age, sex and MRB, with their logarithmic trend lines, in the reference population and plotted MRB of Natufian figurines and cylinders. (Photos and CAD L.D.).

The manufacturing process of these fragments is similar to that of the figurines, suggesting that these artefacts belong to the same techno-symbolic register. However, interpreting them presents several interpretive possibilities.

a. Some are similar to those discovered in Late Natufian Salibiya I (Fig 3E-F; S1 Table) [55] and could be fragments of the bodies of cylindrical figurines (Fig 4.F,H,I). The ‘pillar’ figurines with their cylindrical bodies would later become a significant element in the clay representation of PPNA Gilgal I [58], Jericho [64] or Netiv Hagdud [124], and in Cyprus at Asprokremnos [125]. This mode of figuration subsequently persisted throughout Southwest Asia in the PPNB [55,115] and the PN [126].
b. Some of the fragments can be potentially identified to a specific form. This is the case of a Late Natufian HT fragment (Fig 9C) which profile and curves are reminiscent of those of the anthropomorphic figurine from the same site (Fig 1W) or an Early Natufian EWT fragment (Fig 5 Cb; S18D Fig) which could be a zoomorphic head.
c. Another category of fragments could be figurine appendages. Two of the fragments seem to be the naturalistic depiction of anthropomorphic arms, one from Early Natufian EWT (Fig 5 Cg; S18H Fig) modelled by at least two layers of clay and the other from Late Natufian HT (Fig 5 Be) the triangular end of which recalls the triangular right hand modelled on the Early Natufian figurine from EM (S6G Fig). Another Early or Late Natufian EWT fragment (Fig 8 Em; S18C Fig) could be a naturalistic depiction of the right leg of an anthropomorphic figure depicted in a standing position. The proximal sections of these elements suggest that they were modelled independently before being bonded to a body in an additive process. It should be noted that the dimensions of the probable anthropomorphic appendages of EWT, suggesting that they derive from figurines about 10 cm high, reinforce the hypothesis of a different scale of figuration in this site. Seeing the female figurines from PPNA Mureybet, modelled in standing position [62], and whose schematic legs have been represented by simple cylinders with rounded ends, numerous fragments of the Natufian cylinders (e.g., Fig 5 Aa,Bi,Bk) could also correspond to schematic anthropomorphic legs. Other Late Natufian fragments from HT (S19S Fig) and Early Natufian EM (Fig 5 Af), of a curved conical shape, could be figurations of the horns/antlers of zoomorphic figurines like those seen later in the PPNB [127] and PN [128].

### Use-lives of the figurations

All the artefacts presented here show various degrees of contact use-wear (S4-S17 Figs), suggesting that they formed part of a moving and mobile suite of embodied practices [120]. However, the generally low intensity of this contact use-wear suggests that these manipulations were limited and that the figurines are not objects that were manipulated daily or passed down from generation to generation. Thus, their use-lives were undoubtedly limited, perhaps linked to particular social (e.g., ceremonies) or calendar (e.g., seasonal) events. These parameters undoubtedly correlate with the ease of access to raw materials, the simplicity of the *chaîne opératoire* (S28 Fig), the speed with which it was carried out, and the fragility of the finished products. Similar conclusions were made for the woman/goose figurine from Nahal Ein Gev II (Fig 1X) [46]. In the current state of research, this intensity of use cannot be compared with that of the bone and stone figurations (Fig 1), as almost none of these artefacts have been analysed for use-wear. In any case, the haptic perception [129] and interaction with the observer/user were inevitably different among these three categories of figurations (e.g., different size, robustness, feel, texture, weight and temperature). Indeed, the particular aesthetic format of the clay figurations shows that they are much smaller and more fragile, being made from unbaked clay, than the bone/stone items (e.g., Fig 1W-X compared to the bone and stone figurations of Fig 1). For example, the complete ornithomorphic figurine from the Early Natufian EM (Fig 4F), measuring just 13.7 mm high (Table 1), inevitably implies a close-up viewing creating an intimate contact between the viewer and the figurine. In addition to the size of the figurines, the meticulous incisions made to create their decoration, give volume to the anatomical segments and articulate them smoothly, reinforces this intimate observation by detailed reading of the representation. Furthermore, we did not identify any fittings that would have allowed these figurines to stand freely, implying that they had to be held to be closely observed. This close observation echoes ornamental practices where Natufians strived to invest meaning and convey a different message through barely perceptible variations such as different chromatic variations of red colorant [32], morphometry of beads of the same type, typological associations and the variability of technical treatment [28]. Moreover, in the same way as the ornaments, although the figurations were observed through intimate interactions, they were probably not intended to be observed by a single individual throughout their use-lives. Indeed, given that figurative conventions spread across time and space these figurines were undoubtedly observed by eyes other than those of their makers. The aesthetic format of clay figurations was organised around two different modes of interaction. The first, the most common, concerns figurines modelled around their entire periphery (e.g., Fig 4F), for which complete reading of the image is always delayed since it requires the object to be rotated. The second interaction mode was identified in Final Natufian EM, where two figurines were modelled on one side only (Fig 6 Aa-Ba) to stage the interaction between the observer and the figurine. A similar staging by modeling was identified on the contemporaneous woman/goose figurine from Nahal Ein Gev II (Fig 1X) [46].

### Dermatoglyphic analysis

We identified fingerprints resulting from pressuring and/or pinching gestures during modeling on surfaces of two figurines and five cylinders from all three sites (EWT = 1; HT = 2; EM = 4) (Fig 9). Together with the fingerprints identified on six ornaments and 44 shapings (geometric shapes, nondiagnostic fragments, and non-figurative pellets related to human clay manipulation) from the same sites [31], it forms the largest pre-Neolithic paleodermatoglyphic assemblage known to date. As with the Pavlovian clay figurines [123], this assemblage represents less than 10% of the clay objects discovered at these sites. Our results should, therefore, be interpreted as a significant overview of the Natufian demographics involved in techno-symbolic practices linked to clay. The dermatoglyphic analysis, by measuring the density of epidermal ridges on the fingerprints using the mean ridge breadth (MRB) and comparing it to the experimental dataset of Králík and Novotný [122] (see details in Materials and Methods below) (Fig 9 Ja-Jb), allowed us to establish an overview of the Natufian demographics involved in clay manipulation.

The MRB values of the pooled assemblage (x̄ = 0.404 ± 0.089 mm) (Fig 9 Jb) show a wide distribution, suggesting that, at a regional scale, all categories of the Natufian population (i.e., children to adult males) seem to have been involved in the techno-symbolic interactions with clay. However, there was some difference between the sites since the distribution of their individual MRB (Fig 9 Ja) varies with that of EWT, reaching the highest distribution (x̄ = 0.453 ± 0.108 mm), statistically distinct (t-test p < 0.05) from both HT (x̄ = 0.385 ± 0.045 mm) and EM (x̄ = 0.366 ± 0.072 mm) which are quite similar (t-test p = 0.39).

More specifically, at site level (Fig 9 Ja), ‘children to pre-pubescent sub-adults’ are under-represented at EWT (n = 5; x̄ = 0.346 ± 0.030 mm), and two groups of ‘young adults of both sexes/adult females’ (n = 12; x̄ = 0.420 ± 0.048 mm) and adult males (n = 5; x̄ = 0.609 ± 0.033 mm) are distinct (t-test p < 0.01) (Fig 9 B-Ja). A relatively complete cylinder from Early Natufian EWT (Fig 9A) is covered with multidirectional modeling impressions left by an adult male (MRB of 0.611 mm) (Fig 9 Jc), which raises the question of whether the under-representation of immature individuals at EWT could be linked to the larger size of the figurations modelled there (i.e., larger individuals would have modelled larger figurations). At HT, the demographics are different. Here, adult males are absent, and the prints are those of ‘children to pre-pubescent sub-adults’ (n = 4; x̄ = 0.333 ± 0.029 mm) as well as ‘young adults of both sexes/adult females’ (n = 8; x̄ = 0.411 ± 0.019 mm) (Fig 9 E-Ja). At the interface of these two demographic groups, two individuals (Fig 9 Jc) had left transverse modeling imprints on two cylinders with an elliptical profile of MRB 0.383 mm (Fig 9C) and 0.415 mm (Fig 9D), respectively. In EM, the demographics are similar to HT, adult males are absent, and the fingerprints are those of ‘children to pre-pubescent sub-adults’ (n = 13; x̄ = 0.309 ± 0.037 mm) and ‘young adults of both sexes/adult females’ (n = 11; x̄ = 0.432 ± 0.036 mm) (Fig 9I-Ja). However, here, these two groups are distinct (t-test p < 0.01), and the post-pubescent individuals are represented by higher MRB values than those of HT (i.e., older or of a different sex) (Fig 9 Ja). The back of the Early Natufian EM sub-cylindrical anthropomorphic figurine (Fig 4C; S6 Fig) is marked by two fingerprints (Fig 9F). The first, vertical (Fig 9 Fa), was left by a ‘young adult of both sexes/adult female’ (MRB of 0.456 mm). This fingerprint was then partially covered by a second transverse print (Fig 9 Fb), left by a pre-pubescent child (MRB of 0.345 mm). Such a difference in the density of epidermal ridges cannot be explained by the variability of the fingers from the same individual [130], meaning that these fingerprints belong to two individuals of different ages and possibly sexes. This succession of gestures on the wet clay suggests that, during the modeling sequence, an adult passed on the figurine to a pre-pubescent child. This could be a rare direct testimony of inter-generational knowledge transmission in a hands-on apprenticeship. The Early Natufian EM complete ornithomorphic figurine (Fig 4F; S8 Fig) is marked on its left profile by the transverse fingerprint of a child (MRB of 0.285 mm) (Fig 9 G,Jc), probably under 10 years old. A slightly older child left two successive transverse fingerprints (MRB of 0.310 mm) on a cylinder (Fig 9 Ha). The first is by modeling it (Fig 9 Hb), and then, this print was covered by coloring the cylinder with engobe and a longitudinal smoothing marked with a second print (Fig 9 Hc). On the latter, we can even see apparent features, which can be identified as dotted ridges, a more frequent pattern in children, especially boys [131].

The variation from site to site seems to be related to the age at which Natufians began to interact with clay (Fig 9 Ja) (i.e., children in EM to pre-pubescent children in EWT). Could this be linked to regional variability of socio-cultural constructs based on biological gender (i.e., age and sex)? For example, in the Early Natufian of EM, where children began to interact with clay at an early age, this population category was buried with ornaments more than anywhere else [28]. Be that as it may, it is clear from the HT and EM data that children and pre-pubescent children are not simply curious onlookers who could have touched or handled the figurations before drying or firing. On the contrary, their fingerprints are related to the shaping (Fig 9 G-Hb) and even coloring (Fig 9 Hc) of the artefacts, which suggests that children were involved in the transformation scheme. Clay ornaments marked with fingerprints support this observation [31]. Apart from children, not all categories of adults participated in the same proportions in all sites. Adult males seem absent except in EWT, and the ‘young adults of both sexes/adult females’ category shows high variability between the three sites, probably reflecting the involvement of different individuals among this category (i.e., from young adult males to adult females) (Fig 9 Ja). Thus, we can speculate that the figurative expression in clay had been produced by different age groups in Mount Carmel (EWT), the Galilee (HT) and Upper Jordan Valley (EM). It also appears that the community of practice was multigenerational. Similar conclusions were reached for the Upper Palaeolithic Pavlovian clay figurative tradition [71] based on the analysis of 48 shaping fingerprints (Fig 9 Jb) [96,123].

Different models identified in ethnographical and archaeological records [e.g., 132] could perhaps explain this multigenerational community of practice. In many more recent cultures, where adults produce pottery, children produce, at the same time, miniature models [133]. The result is an assemblage of anthropomorphic and zoomorphic figurines, vessels, tools and even architecture, capturing their society’s economic, ideological, and cultural traditions [134]. The production of these miniature objects is part of two distinct socialisation processes, related to kin and non-kin. Either the modeling was done through play and apprenticeship by observing and imitating peers groso modo [e.g., 135] and through observing or learning from adults [136]. These two venues of practice, which can be simultaneous in some socio-economic frameworks, result in two distinct ways children socialise through engagement with clay [132]. In the first instance children develop open skills through learning that allows experimentation and failure creating freely, in various forms [e.g., 137]. Ethnoarchaeology has shown that in some cases, the learning of how to model figurines, including the steps of clay preparation, shaping, and firing, was not to produce figurines per se but to serve as an activity for children to play constructively together and build a community [138]. Conversely, in a relationship where the production of miniature objects prevails over peer socialisation, apprenticeship with adults emphasises fidelity to traditional formats. It encourages precise imitation that promotes the development of closed skills and regularity in production. This apprenticeship can be seen as an early step in obtaining the handiness needed to make full-scale, ‘adult tradition’ artefacts [139].

As for the Natufian, we will refrain from making a hypothesis of how those artefacts were produced given that at this stage we only observe the tip of this rich clay figurative tradition. However, if we focus on the ornithomorphic figurations from EM, which demonstrate the fidelity to a figurative convention for thousands of years, it seems likely that, in this case, children were most probably guided by an ‘adult tradition’. Therefore, these artefacts could be interpreted as portable figurative mnemotechnic devices [110] related to children’s learning of traditional beliefs and ethno-ornithological knowledge.

### Spatial distribution

Though portable figurative expression emerged within the Natufian settlements and thus concomitantly with a new relationship to space engendered by sedentism, little is known about the spatial organisation of figurative expression. Indeed, as Cauvin [62] noted, the rare Natufian figurations were mostly discovered in isolation, deprived by the chances of their deposition of spatial relationship, of contiguities or reciprocal exclusions that could allow to deduce a significant typo-stylistic arrangement and specific thought structures. Early Natufian Wadi Hammeh 27 marks an exception as an association between zoomorphic and phallic representations is noted in two successive occupation phases [25,40,41]. The discovery of Natufian clay figurations changes the picture, as the figurative artefacts are now numerous enough to allow analysing their spatial organisation in the domesticated space. Our analysis will be limited to EM, as this is the site in which the artefacts are most numerous and the areas excavated the most extensive. Here, we concentrate on two sectors, in the Early and Final Natufian (see details in the Archaeological Methods section) that enable us to follow the evolution of the spatial distribution of techno-symbolic clay practices between the beginning and the end of the Natufian occupation. During the Early Natufian (Fig 10A), floor IVa of Shelter 131 was only briefly occupied, abruptly abandoned, and quickly covered [140]. These conditions made it possible to preserve thousands of *in situ* artefacts relating to both domestic and spiritual activities, leading F. Valla [19] to suggest that in the early stages of the Natufian, there was no thinking of a clear dichotomy between the mundane and the spiritual in EM. Among the evidence of symbolic activities are associated deposits of fauna and human remains (i.e., gazelle horn core, a dog mandible and a skullcap) [140], body decorations made from biogenic materials [28] and clay [31], colorful manuports, stone figurative (Fig 1N) and non-figurative artefacts [19]. We can now add to these four figurines and six clay cylinders (Fig 10A). From this assemblage, we can see that all the clay figurations (i.e., anthropomorphic, ornithomorphic and cylindrical) coexisted in their use and perhaps their manufacture without spatial restriction inside the habitat. These rapidly manufactured items, some of them produced by children (Fig 9F-G) and in readily available material, are found side by side with a calcite zoomorphic figurine (Fig 8 Ab), indicating a high degree of transformation, technical investment and made in an exogenous material. Moreover, the density of this clay assemblage, in the light of the small area of floor space preserved, suggests that these practices were not episodic and that they could represent the predominant mode of figurative expression in Shelter 131.

**Fig 10:**
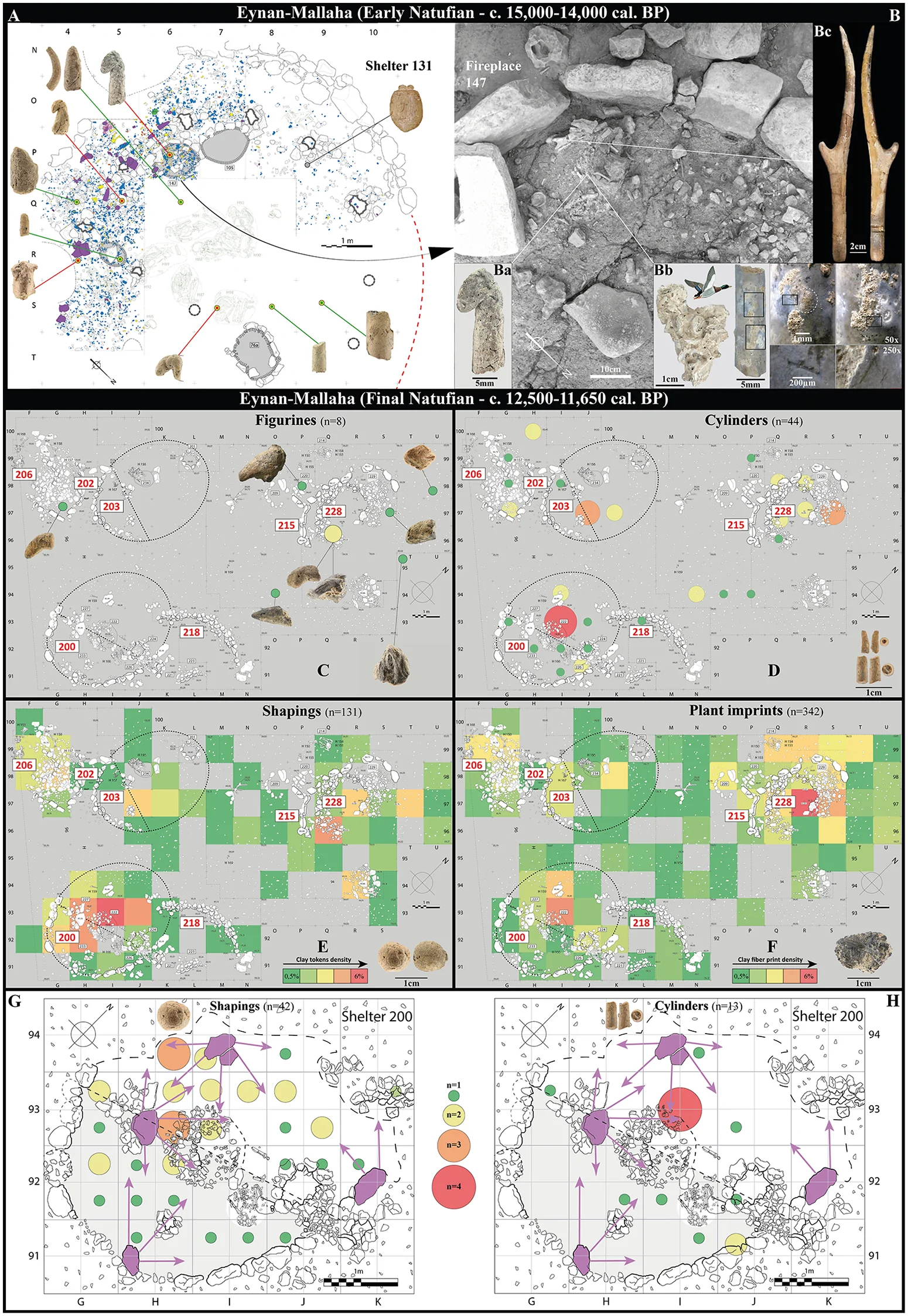
Spatial distribution of clay artefacts in EM. Early Natufian, Shelter 131 – Floors IVa (A) Plan with the position of figurines (in red, n = 4), cylinders (in green, n = 6) and the calcite figurine on the floor; (B) Zenithal photo of the fireplace 147 inside which (Ba) the ornithomorphic figurine EM76 4562 was discovered among (Bb) Calcinated and ash cemented Mallard bones (*A. platyrhynchos*) of which the wing-feathered bones show cutmarks suggesting feather extraction (see S26 Fig); (Bc) A roe deer antler prestige object (EM75 4147.420) intentionally broken and discarded in the fireplace. Final Natufian, Layer Ib (shelters numbers written in red, boundaries indicated for shelters 200 and 203: the south delimited part is the roofed part): (C) Position (by 1x1 square) of figurines indicated by dots (n = 8); (D) Position (by 1x1 square) of cylinders indicated by dots (n = 44); (E) Density (%/m²) of shapings (n = 131); (F) Density (%/m²) of clay objects with vegetal imprints (n = 342). Detail of Shelter 200 (south roofed part in grey) with the position (by sub-square) of the clay artefacts compared to the identified activity stations and their areas of diffusion (in purple) [78]: (G) Shapings (n = 42); (H) Cylinders (n = 13). (Photos F.R.V., L.D. and CAD L.D.).

Contrary to the Pavlovian clay figurines [96], here not all clay artefacts are concentrated in or around fireplaces. Note the case of the cylindrical ornithomorphic figurine, probably representing an anatid bird (Fig 10 Ba), discovered in the ash fill of Fireplace 147 (Fig 10B), the centre of 131 dwelling, which attracted most of the technical, culinary and ritual activities [19,140]. More precisely, this duck-like figurine was discovered among an exceptionally dense concentration (c. 500 NISP) of calcinated and ash-cemented Mallard bones (*Anas platyrhynchos*) [100] of which the wing-feathered bones show cutmarks around the remigial papillae (i.e., where the feathers attach to the bone) (Fig 10 Bb; S26 Fig), suggesting feather extraction. This practice, probably linked to personal ornamentation and identified also in Late Natufian Nahal Ein Gev II with Greylag goose feathers (*Anser anser*) [46], was observed in other areas of Shelter 131 and on other species of birds. However, its concentration in Fireplace 147 is unique and attests to the importance of the Mallard in the symbolic sphere of the inhabitants of Shelter 131. In this way, the physical association of a duck-like clay figurine with Mallard bones, which it was perhaps intended to represent, is worthy of attention. On the other hand, we might wonder why the figurine was in the fireplace in the first place. Is it a figurine lost/forgotten there after being deliberately baked? Had it been thrown into the fireplace to be destroyed in a ritual pyrotechnical performance similar to what was conjectured for the Pavlovian figurines [72]? We can note that traces of red colorant and contact use-wear (S9B,C,F Fig) suggest that the figurine was handled and colored before being placed in the fireplace. Still, the long vertical fracture along the neck/body cylinder (S9 Fig) might be interpreted as a crack typical of thermal shock, indicating that the figurine was fired before it was sufficiently dry. We could, therefore, hypothesise that the figurine was thrown into the fireplace after being broken by poorly controlled firing. The hypothesis of ritual pyrotechnical destruction also makes sense when we consider that this is the fate of a large prestige object made from a cervid antler discovered fragmented among the calcinated Mallard bones (Fig 10 Bc) [19,88]. A future analysis of these different objects’ heating temperatures might help to verify one of these hypotheses.

The Final Natufian occupation (layer Ib) (Fig 10C-H) is circa 50 cm thick, over an excavated surface of about 120 m^2^ and includes five distinctive stone shelters distributed into two temporally-separated building phases [141]. Here, the larger size of the clay assemblage (n = 525) makes it possible to compare the spatial distribution of the complete typological variability of clay artefacts (figurines, cylinders, geometric shapes, non-diagnostic fragments, non-figurative pellets and plant imprints). All were discarded with many other forms of occupational waste (see [78] for detailed spatial distribution and identified activity stations). The distribution of the eight clay figurines attributed to this phase is shown in Fig 10C. To the south, no figurines were discovered in dwellings 200 and 203, and just a fragment of an ornithomorphic head (Fig 4G; S12 Fig) was found in the specialised shelter 202/206, dedicated to pyrotechnic activities [78]. To the north, another ornithomorphic head fragment (Fig 4K; S15 Fig) was recovered (Sq.O94) deeper in the slope of the ‘stony layer’, where much occupational waste had been discarded. The majority of figurines, whether anthropomorphic (Fig 4D), ornithomorphic (Fig 4L,O), or zoomorphic (Fig 4A,B), was concentrated in the shelter 215/228 and the working area outside it. Two head fragments, zoomorphic (Fig 4A) and ornithomorphic (Fig 4O), were discovered in the same stratigraphic horizon. The only anthropomorphic figurine (Fig 4D) was discovered at the boundary of the excavation sector (Sq.T95), identified as a possible trash area [84]. The concentration of figurines in the shelter 215/228, which is not a dwelling, but a specialised structure linked to cooking [84], is remarkable. Indeed, it is notable that here, the figurines representing animals are concentrated in the architectural complex where the hunters brought their prey to be cooked. So, as in the Early Natufian, the different subjects depicted seem to coexist in the same architectural ensemble. However, by the end of the Natufian, figurines seem absent from dwellings (shelters 200 and 203) and concentrated in specialised structures (shelters 202/206 and 215/228). A similar trend was observed in the spatial distribution of clay ornaments [31].

The distribution pattern of cylinders (n = 44) (Fig 10D) differs and could depict a variability in either use, function or discard of different types of figurative artefacts. Cylinders have been discovered in dwellings 200 and 203, mainly concentrated in their northern non-roofed part (Fig 10H). This trend is noteworthy because it is the opposite of what we see for wing-feathered bones of Anatinae that were curated in the southern, roofed part, of these dwellings [34] where possible bedding areas have been isolated [78]. The same quantity of cylinders (n = 20) was found in the specialised structures 202/206 and 215/228 (Fig 10D), which suggests that their use, function and/or discard is not restricted to dwellings. Only four cylinders have been discovered in the ‘stony layer’ outside the built structures, which suggests that their use, function and/or discard was concentrated in the domestic spaces. A comparison of the two larger assemblages, that from dwelling 200 (n = 14) and that from specialised shelter 215/228 (n = 14), reveals that the cylinder fragments are significantly larger (t-test p < 0.05) and more red-colored in the specialised shelter. It is hard to interpret these differences, as they could be explained by differential conservation of the two contexts.

The spatial distribution of shapings (n = 131) (Fig 10E) echoes that of the cylinders. There is a variation between dwellings since shelter 200 (n = 43) is much richer than shelter 203 (n = 15), a trend similar to that observed for the curated wing-feathered bones of Anatinae [34]. It thus seems safe to state that task specialization and differential use of space occurred in the symbolic practices. The variation between the two dwellings is probably due to the presence, only in Shelter 200, of a central fireplace (St.222), which acted as an attractive hub of activity [78]. Indeed, the peripheral fireplaces did not (St.201 in Shelter 203) or very little (St. 224 in Shelter 200) ‘attract’ clay objects. Apparently, the clay handling was mainly concentrated in the open area in Shelter 200 (Fig 10G), spreading from the activity stations around the central fireplace. Shapings are evenly distributed between the habitats (44.3%; n = 58 in Shelters 200 and 203) and specialised shelters (41.2%; n = 54 in Shelters 202/206 and 215/228), which suggests that their use, function and/or discard is not related to the function of the architectural ensemble *per se*.

Finally, it is difficult to interpret the spatial distribution of the clay objects with plant imprints (n = 342) (Fig 10F). The status of this corpus is uncertain, as it is difficult to establish whether it should be associated with the techno-symbolic sphere. Whatever the case, we can be sure that these objects bear witness to the manipulation of wet clay, which was then heated, whether intentionally or not. Therefore, we will interpret it as background noise that can shed light on the spatial variability and intensity of clay manipulation. Here, the signal is clear since the dwellings (Shelters 200 and 203) account for only a quarter (27.8%; n = 95) of the clay objects with plant imprints, while nearly half of them (45.3%; n = 155) are concentrated in and around the specialised shelter 215/228. Future petrographic analyses may shed light on the relationship between objects with plant imprints and the chaîne opératoire of clay techno-symbolic artefacts.

We can observe evolutionary trends in the spatial distribution of techno-symbolic clay practices between the beginning and the end of the Natufian occupation at EM. While during the Early Natufian, all the practices seem to be attested in dwelling 131, the situation changes drastically in the Final Natufian when specialised shelters ‘host’ a significant number of clay figurines. The distribution of all the artefacts associated with figuration (e.g., figurines, cylinders and shapings) is quite extensive (S27 Fig). This spatial dispersal is accompanied by the partitioning of symbolic practices within the dwelling structure. Anatid wing bones are concentrated in the covered part of the habitat [34], while the clay is handled in the open area. The combined analysis of the spatial distribution of other techno-symbolic traditions in EM as well as other Natufian settlements will undoubtedly allow us to refine our understanding of these practices.

## Discussion

The 154 Natufian clay figurative artefacts presented here, produced in multigenerational communities of practices and figurative conventions across three major settlements of the Southern Levant, are the earliest evidence of a figurative clay tradition in Southwest Asia where it marks an important step in the evolution of human technology. For the first time in this region, the figurative practice did not consist of using or reducing a pre-existing natural preform but of constructing and modeling a shape in a plastic material following a mental template. This shift required diverse tools, skills and knowledge, different from those needed for the manipulation of stone and bone. The findings reported here, together with the clay ornamental tradition in the same sites [31] and the woman/goose figurine from Nahal Ein Gev II (Fig 1X) [46], challenge the longstanding regional paradigm associating the emergence and development of the symbolic use of clay in tandem with the Neolithic productive economy and its subsequent social, symbolic and technological innovations. The Natufian figurative clay tradition is also challenging the association between the Neolithic transition of Southwest Asia and the emergence of new symbols (i.e., ornithomorphic), new figurative techniques (i.e., engobe coloration and composite figurines), and new figurative practices (i.e., iconicity). This discovery has changed our perception of Natufian figurative expression, which was much more intense and diverse than previously perceived. This intensity also contrasts with the figurative expression of the Early Neolithic, given that the size of the assemblage presented here, from only three sites, is already larger than that of all the PPNA clay figurations known today from the whole Levant. Through a simple chaîne opératoire (S28 Fig), meticulously carried-out by multigenerational manufacturers, clay has become a primary medium of figurative and perhaps even non-figurative expression (i.e., geometric and abstract motifs) (Fig 8D) to serve a variety of functions in a variety of domestic contexts.

Clearly, there is the emergence of narrative figuration as early as the Early Natufian, which transcribed animals in courtship behaviours, emphasising movement and giving the figures an expression of being animate. This figurative shift in zoomorphic expression reflects developments through deep observation and accumulation of practical knowledge, possibly stored and transmitted in ritualised performance [110]. It predates by three millennia the stone figures of the monumental cult buildings at PPNA Göbekli Tepe [142]. There and in other PPNA sites of Upper Mesopotamia, it was interpreted as a marker of ontological change, documenting how the Early Neolithic study of nature reached a new quality and the “beginning perception of Neolithic communities as not just being part of nature, but becoming actors capable of manipulating and transforming their environment” [143]. More than ever, it places the symbolic, cognitive and ontological foundations of the Neolithic transition in the Natufian culture, with many techno-symbolic innovations previously thought to be exclusively linked to Neolithic societies. In contrast to Europe [70–73] and North Africa [74, 75], where similar Palaeolithic clay innovations remained isolated, were not continued, in Southwest Asia, Natufian developments have shaped figurative expression for millennia.

Our renewed chronology of techno-symbolical developments suggests that the concepts of figurative and narrative expression, with the ontological and technical means of their implementation on bone, stone and clay, emerged at least in the Early Natufian (c. 15,000 cal. BP), at the same time as the sedentary lifestyle. This unprecedented transformation of symbolic systems in the chrono-cultural sequence of Southwest Asia, had been probably linked to the evolution of the sedentary communities’ ontological filters conditioning new interactions with their social and natural environments [106,144]. It was not recognised by Jacques Cauvin in his search for the symbolic, cognitive and ontological foundations of the Neolithic transition [62]. While acknowledging the near absence of figurative expression prior to the Natufian, Cauvin considered that “the art of the last Natufian hunter-gatherers has taught us little except that it was essentially zoomorphic” [62]. Therefore, he recognised the first evidence of the ‘revolution of symbols’ in the beginning of the Early Neolithic in the Khiamian culture (PPNA c. 11,650–11,500 cal. BP), by identifying a change from the Natufian in figurative expression, the “spectacular humanisation” of the representations and the emergence of feminine figurines [62]. Natufian anthropomorphic figurations on bone (Fig 1 Aa), stone (Fig 1O-V) and clay (Fig 4C-D) including feminine figurines (Fig 1W-X), as well as the diffusion of Natufian ornithomorphic figurative repertoire (Figs 1I and 4E-O) to the Early Neolithic [e.g., 79] (S25 Fig), challenge the ‘spectacular’ nature of the change observed by Cauvin, which seems to be more of a step than a starting point. Above all, it seems essential to reconsider the transformation of symbolic systems initiated by the Natufian communities and the sedentary lifestyle to interpret it as the starting point of the ‘revolution of symbols’ at the origin of the Neolithic transition of Southwest Asia.

Our understanding of the spatio-temporal variation of these practices can be developed through several lines of research. Identifying clays by petrography and colorants by archaeometric techniques will enable us to clarify their acquisition patterns. Identifying the heating modes will enable us to shed light on the role of pyrotechnology and the possible emergence of ceramic technology (i.e., clay intentionally hardened by intentional heat treatment) in Southwest Asia. Using archaeobotany to characterise the decorative plant imprints will shed light on the possible symbolic role of certain plants. Furthermore, to characterise the conditions of the emergence of the symbolic use of clay in the Natufian and measure the impact of sedentarisation on these, it seems essential to investigate the more mobile cultures of the Early and Middle Epipalaeolithic to detect any early beginnings of clay manipulation. Finally, this discovery might stimulate further research into early clay manipulations in Southwest Asia by reevaluating previously unstudied collections of clay lumps.

## Materials and methods

### Archaeological methods

#### el-Wad Terrace (EWT).

The site of el-Wad, comprising a large cave and an adjacent terrace with a rich and long Natufian sequence spanning most of the duration of this cultural entity (c. 15,000–11,650 cal. BP), is located at the foot of Mount Carmel, Israel, facing northwest towards the Mediterranean coastal plain (Fig 2) [21,66]. All the material studied and reported herein originated in el-Wad Terrace’s Early and Late Natufian layers excavated, on a surface of c. 50 m², by M. Weinstein-Evron, D. Kaufman, and R. Yeshurun from 1994 until present (see a detailed description in [21]) on behalf of the University of Haifa (Excavation permits G-44/1994 to G-8/2023 issued by the Israel Antiquities Authority). The area was divided to grid squares, each 1 m², which were further subdivided to subsqaures (0.25 m²). Each subsquare was excavated to a depth of 5 cm or less, creating our basic volumetric unit (“basket”). Special phenomena such as architectural features, sediment patches and concentration of finds were designated as loci and excavated accordingly, maintaining the same grid system. Stones larger than 10 cm and outstanding artefacts were mapped to scale. All sediments were saved; a large portion sorting to retrieve delicate findings such as charcoal pieces, and all sediments were wet-screened with 5 and 1 mm meshes.

#### Hayonim Terrace (HT).

The site of Hayonim, comprising a large cave [e.g., 24, 145] and an adjacent terrace, with a rich and long Early and Late Natufian occupations, is located in Western Galilee, Israel, facing the Nahal Meged (Fig 2). All the material studied and reported herein originated in Hayonim Terrace’s Late Natufian (Layer II) excavated, on a surface of 32 m², in 1980, 1981 and between 1985 and 1989 by F.R. Valla (c. 13,000–11,650 cal. BP – see a detailed description in [146]) on behalf of the Centre de recherche français à Jérusalem (Excavation permits G-17/1980 to G-53/1989 issued by the Israel Antiquities Authority). Generally speaking, the excavation was conducted using a unit system of a quarter square meter (0.25 m²), 5 cm deep. This system was adapted according to the findings. The undifferentiated surface layer was excavated using a 1 m² grid, shifting to the quarter square meter grid inside and outside structures as soon as they were identified. The fill of each structure was isolated, introducing ‘natural’ boundaries instead of the artificial limits established by the grid. Nevertheless, the artificial grid subdivisions were respected in each structure to allow spatial distribution of the small finds. The thickness of the units was also adapted to the stratigraphy, resulting in units much thinner than 5 cm, especially when searching for ‘floors’ and surfaces. Inside the structures, all items larger than 5 cm (bones, stones, lithic artefacts and other finds) were mapped. Sediments were wet-sieved using a 1–2 mm mesh and further sorted in the laboratory.

#### Eynan-Mallaha.

The 2000 m² open-air site, in the Hula Lake Basin of the Upper Jordan Valley, Israel (Fig 2), is known for its rich Natufian deposits spanning most of the duration of this cultural entity (c. 15,000–11,650 cal. BP – see a detailed description in [20]). All the material studied and reported herein originated in Eynan-Mallaha’s Early (Layers IV-II), Late (Layer Ic) and Final (Layer Ib) Natufian deposits excavated by J. Perrot, M. Lechevallier and F.R. Valla between 1955 and 1976 on behalf of the Centre de recherche français à Jérusalem (Excavation permits &-35/1955 to A-849/1979 issued by the Israel Antiquities Authority); F.R. Valla and H. Khalaily between 1996 and 2005 (see a detailed history of excavation in [20]) on behalf of the Centre de recherche français à Jérusalem and Israel Antiquities Authority (Excavation permits G-829 81/1996 to G-51/2005 issued by the Israel Antiquities Authority). Generally speaking, the excavation was conducted using a unit system of a quarter square meter (0.25 m²), 5 cm deep. This system was adapted according to the findings. The undifferentiated surface layer was excavated using a 1 m² grid, shifting to the quarter square meter grid inside and outside structures as soon as they were identified. The fill of each structure was isolated, introducing ‘natural’ boundaries instead of the artificial limits established by the grid. Nevertheless, the artificial grid subdivisions were respected in each structure to allow spatial distribution of the small finds. The thickness of the units was also adapted to the stratigraphy, resulting in units much thinner than 5 cm, especially when searching for ‘floors’ and surfaces. Inside the structures, all items larger than 5 cm (bones, stones, lithic artefacts and other finds) were mapped. Sediments were wet-sieved using a 1–2 mm mesh and further sorted in the laboratory. The sieving of excavated sediments was not systematic before the discovery of the floor of Shelter 131 in 1975 [147].

### Natufian clay figurations analysis

Clay artefacts were identified by sorting tens of thousands of small (mostly less than 2 cm^3^) heated clay and sediment lumps (c. 40 kg in total) of different shapes and colors (S2 Table and S1 Fig). These collections were formed by the sieving and sorting of excavated sediments from specific contexts encompassing the whole duration of the Natufian culture. All the available heated clay and sediment lumps collections from EWT, HT and EM were sorted, ensuring that all sieved contexts were investigated equally by the present study. All necessary permits were obtained for the described study, which complied with all relevant regulations. The figurations were analysed microscopically with a Dino-Lite AD-7013MZT digital microscope (magnification 30–250×) and pictures were taken with a Canon 7D camera (180 mm Tamron Macro lens) with Helicon Remote and Helicon Focus softwares (https://www.heliconsoft.com/) at the Centre de recherche français à Jérusalem, the Hebrew University of Jerusalem and the University of Haifa. Technical traces on the artefacts were measured using high-resolution pictures imported into Image J software (https://imagej.net/ij/). Digital image enhancement was done with DStretch (https://www.dstretch.com/). CAD (computer-aided design) was done with Adobe Illustrator 2025.

### Scanning electron microscopy – energy dispersive spectroscopy (SEM–EDS)

SEM-EDS was implemented for the determination of residues identified on the surface of the clay figurations. Micro analyses were performed on a Cryo High-Resolution Scanning Electron Microscope Apreo 2S at the Center for Nanoscience and Nanotechnology (Unit for Nano Characterization) of the Hebrew University of Jerusalem.

### Dermatoglyphic analysis

The fingerprints were identified at macroscopic observation and the Mean Ridge Breadth (MRB) was measured using high-resolution pictures imported into Image J software. Following the method suggested in the developing field of archaeological dermatoglyphics [148, 149], to estimate the age of the manufacturer, the Natufians MRB were compared to the modern experimental dataset of Králík and Novotny [122]. It was measured on the fingerprints left on clay artefacts modelled by 118 individuals aged 1–77 years old (44 males and 74 females) from the Czech Republic and allowed to delimit three demographic categories (children to pre-pubescent sub-adults <15 years old MRB < 0.39mm; young adults of both sexes and adult females MRB > 0.39 to <0.52mm; adult males MRB > 0.52mm) [122]. This comparison is possible given that despite the wide variation between worldwide populations [150], the ratio between average epidermal ridge densities and age and sex remains constant across populations [149]. While one has to consider the shrinkage of the fingerprints in the drying and firing stages of the clay artefacts, which was likely between 2% and 6% [151], it does not negate the results of the comparison. In this preliminary dermatoglyphic study, we have limited our estimations to the age, but not sex, of children and the sex, but not age, of adults (i.e., MRB values for adult females and young adult males are inherently indistinguishable at this stage). To increase the accuracy of our estimations, in the second stage, we will develop experimental reference datasets and apply the Bayesian mixture model developed by Burchill and colleagues [148].

## Supporting information

S1 Fig Example of heated clay and sediment lumps of different shape and color from the Final Natufian of Eynan-Mallaha (Photos L.D.). (TIF)

S2 Fig Box plot and Jitter plot of the morphometric variability of Natufian clay cylinders from el-Wad Terrace (EWT), Hayonim Terrace (HT) and Eynan-Mallaha (EM). (TIF)

S3 Fig Natufian clay cylinders from el-Wad Terrace, Hayonim Terrace and Eynan-Mallaha, A: Variability of the general shape of the fragments; B: Variability of the fragment’s broken end shape; C: Variability of the fragment’s preserved end shape. (TIF)

S4 Fig Fragmented clay zoomorphic figurine EM97 6140 (Final Natufian). Aa, Ba, Ca: Residues of red colorant on different points of the figurine’s head; Ab, Bb, Cb: Contact use-wear on different protruding points of the figurine’s head (Photos L.D.).
(TIF)

S5 Fig Fragmented clay zoomorphic figurine EM99 7121 (Final Natufian). A: Engobe layer on top of the clay matrix forming the figurine’s head; B&C: Contact use-wear on protruding points of the figurine’s hears; D: Clay matrix forming the figurine’s head under the engobe; E&G: Contact use-wear on protruding points of the top and bottom of the figurine’s head; F: Blunt tool incision (U section c. 350 µm wide) shaping the bottom of the left hear (Photos L.D.).
(TIF)

S6 Fig Fragmented clay anthropomorphic figurine EM75 4551.17 (Early Natufian). A: Blunt tool incisions (U section c. 350 µm wide) marking the limit between the left arm and the chest; B&C: Contact use-wear on a protruding point of the left elbow; D&E: Impressions made in the wet clay of the right side of the chest by using the articular end of a bird or small mammal bone (e.g., distal end of a phalange) (c. 2,3 mm wide); Fa: Contact use-wear on a protruding point of the right shoulder; Fb: Blunt tool incisions (U section c. 350 µm wide) on the right elbow; G: Contact use-wear on a protruding point of the right hand; H: Series of at least nine vertical and parallel shallow blunt tool incisions (U section c. 500 µm wide) under the right arm; I: Contact use-wear on protruding points of the broken neck (Photos L.D.).
(TIF)

S7 Fig Anthropomorphic clay figurine EM01 8745.6 (Final Natufian). A: Blunt tool incisions (U section c. 250 µm to 450 µm wide) and contact use-wear on the left side; B: Upper face notch where a pointed perishable material was inserted vertically in the clay; C: Shallow imprints of a flat vegetal? tool (c. 200 µm wide) used to shape the figurine’s left eye; D: Contact use-wear on the protruding part of the figurine’s nose; E: Residues of red colorant on the surface; F: Detail of the parallel curved lines pattern shaped in the clay; G: Contact use-wear on the protruding part of the figurine’s distal part; H: Contact use-wear on the edges of the upper face notch (seen from the top) where a pointed perishable material (outer diameter of 1,6 mm) was inserted vertically in the clay; I: Upper face notch seen from the back (Photos L.D.) (Drawing Artist Credit: Dasha Lokshin, Haifa University).
(TIF)

S8 Fig Clay ornithomorphic figurine EM75 4585 (Early Natufian). Aa: Contact use-wear on a protruding point of the left side of the head; Ab: Shallow imprints of a flat vegetal? tool (c. 350 µm wide) used to shape the figurine’s head; B: Residues of red colorant on the left side of the figurine’s head; C: Four blunt tool incisions (U section c. 200 µm wide) marking the limit between the head and the cylinder neck/body; D&E: Contact use-wear on protruding points of the distal section of the front face; F: Contact use-wear on a protruding point of the right side of the head; G: Contact use-wear on a protruding point of the back face; H: Bone splinter embedded in the clay; I: Partial fingerprint on the top of the head (Photos L.D.).
(TIF)

S9 Fig Fragmented clay ornithomorphic figurine EM75 4562 (Early Natufian). A&D: Sharp tool incisions (V section c. 250 µm wide) marking the limit between the head and the cylinder neck/body; B: Contact use-wear on protruding points of the distal section of the left face; C: Contact use-wear and residues of red colorant on the front of the figurine’s head; E: Possible iron-rich clay in the cylinder neck/body; F: Contact use-wear on a protruding point of the distal section of the right side (Photos L.D.).
(TIF)

S10 Fig Fragmented clay cylinder figurine EM74 3399 (Early Natufian). A: Bone splinter embedded in the clay; B: Blunt tool incision (U section c. 250 µm wide) marking the limit between the head and the cylinder neck/body; C: Detail of the stencilled twisted line engobe decoration; D: charcoal and bone splinter embedded in the clay; Ea: Shallow imprints of a vegetal? shaping tool (c. 300 µm wide); Eb: Contact use-wear on a protruding point; F: Contact use-wear on a protruding point and quartz inclusions in the clay; G&H: Charcoals embedded in the clay (Photos L.D.).
(TIF)

S11 Fig Head fragment of an ornithomorphic clay figurine EM74 3339 (Early Natufian). A: Blunt tool incision marking the limit between the head and the beak; B: Shallow transversal marks left by a shaping tool under the head; C: Contact use-wear on the beak and charcoal embedded in the clay; D: Charcoal embedded in the clay on the right side; E: Contact use-wear on the protruding part of the figurine’s head (Photos L.D.).
(TIF)

S12 Fig Head fragment of an ornithomorphic clay figurine EM05 9803 (Final Natufian). A: red colorant on the right side of the head; B: red colorant and contact use-wear on the bottom side of the head (Photos L.D. - Magnification 220x).
(TIF)

S13 Fig Head fragment of an ornithomorphic clay figurine EM97 5828 (Final Natufian). A&B: Residues of red colorant on the right side of the figurine’s head; C: Contact use-wear on a protruding point of the figurine’s head top; D: Blunt tool incision (U section c. 250 µm wide) used to shape the profile of the head; E: Contact use-wear on a protruding point of the figurine’s head bottom (Photos L.D.).
(TIF)

S14 Fig Head fragment of an ornithomorphic clay figurine EM97 6104 (Final Natufian). Contact use-wear on different protruding points of the figurine’s head (Photos L.D.).
(TIF)

S15 Fig Head fragment of an ornithomorphic clay figurine EM98 6600 (Final Natufian). Residues of red colorant and contact use-wear on different protruding points of the figurine’s head (Photos L.D.).
(TIF)

S16 Fig Head fragment of an ornithomorphic clay figurine HT86 1272 (Late Natufian). A: Blunt tool incision (U section c. 250 µm wide) marking the limit between the head and the beak on the left side; B: Residue of brown/black matter applied transversally on the clay possibly to figure the left eye; C: Contact use-wear on a protruding point of the figurine’s head; D: Charcoal embedded in the clay matrix; E: Parallel longitudinal grooving marks left by a flat tool (c. 400 µm wide) possibly to shape the right eye; F: Contact use-wear on the figurine’s beak; G-K: Bone embedded in the clay matrix; L: Contact use-wear and transversal shaping marks under the figurine’s beak; H&I: Contact use-wear and longitudinal shaping marks left by a flat tool; J: Blunt tool transversal incisions (U section c. 250 µm wide) shaping the figurine’s beak on the right side (Photos L.D.).
(TIF)

S17 Fig Head fragment of an ornithomorphic clay figurine HT86 1632 (Late Natufian). A-C-Ea-Eb: Contact use-wear on protruding points of the figurine’s head left side; B-H-L: Blunt tool incisions (U section c. 600 µm wide) marking the limit between the head and the neck/body; D: Blunt tool incision (U section c. 500 µm wide) on the back of the figurine’s neck; Ec-J-M-P: Residues of red colorant on different points of the figurine’s head; F: Bone embedded in the clay matrix; G-I-K-N-O-Q: Contact use-wear on protruding points of the figurine’s head (Photos L.D.).
(TIF)

S18 Fig el-Wad Terrace (Early – Late Natufian) Clay cylinders fragments (A-K) (Photos L.D.). (TIF)

S19 Fig Hayonim Terrace (Late Natufian) clay cylinders fragments (A-V) (Photos L.D.). (TIF)

S20 Fig Hayonim Terrace (Late Natufian) clay cylinders fragments (A-K) (Photos L.D.). (TIF)

S21 Fig Hayonim Terrace (Late Natufian) clay cylinders fragments (A-I) (Photos L.D.). (TIF)

S22 Fig Eynan-Mallaha (Early to Final Natufian) clay cylinders fragments (A-U) (Photos L.D.). (TIF)

S23 Fig Eynan-Mallaha (Early to Final Natufian) clay cylinders fragments (A-U) (Photos L.D.). (TIF)

S24 Fig Eynan-Mallaha (Early to Final Natufian) clay cylinders fragments (A-K) (Photos L.D.). (TIF)

S25 Fig PPNA stone ornithomorphic figurines from A: Mureybet ([89], courtesy of D. Stordeur); B: Jerf el-Ahmar ([90], courtesy of D. Stordeur); C: Göbekli Tepe [99]; D: Gilgal I [58]; E: Körtik Tepe ([94], courtesy of V. Özkaya); F: Nemriq 9 ([91, 92], courtesy of R.F. Mazurowski).
(TIF)

S26 Fig Eynan-Mallaha, Early Natufian, Shelter 131 – Floors IVa and IVb - Fireplace 147. A fragmented ulna (EM75 4147), from the c. 500 calcinated and ash cemented Mallard bones (*Anas platyrhynchos*) discovered in the fireplace, that show cutmarks around the remigial papillae (encircled in dotted lines), suggesting feather extraction (Photos L.D.).
(TIF)

S27 Fig Spatial distribution of clay figurations (figurines, cylinders and shapings) in the Final Natufian, Layer Ib of Eynan-Mallaha (shelters numbers written in red, boundaries indicated for shelters 200 and 203: the south delimited part is the roofed part). (CAD L.D.).
(TIF)

S28 Fig Diagram illustrating variations of the chaîne opératoire of Natufian clay figuations (CAD L.D.). (TIF)

S1 Table List of Late Epipaleolithic clay artefacts previously identified in the Levant and Anatolia. (XLSX)

S2 Table List of the sites, weight of clay lumps sorted, and inventory of artefacts included in this study. (XLSX)

S3 Table Inventory of the Natufian clay cylinders from el-Wad Terrace (EWT), Hayonim Terrace (HT) and Eynan-Mallaha (EM). (XLSX)

S4 Table List of stylistic characteristics (when observable) of the Natufian clay ornithomorphic figurines from Hayonim Terrace (HT) and Eynan-Mallaha (EM) included in this study. (XLSX)

S5 Table Morphometric variability of Natufian clay cylinders from el-Wad Terrace (EWT), Hayonim Terrace (HT) and Eynan-Mallaha (EM). (XLSX)

S1 Movie Rotating 3D photogrammetry (right profile view) of the clay zoomorphic figurine EM97 6140 (Fig 4A – Final Natufian). (MP4)

S2 Movie Rotating 3D photogrammetry (top view) of the clay zoomorphic figurine EM97 6140 (Fig 4A – Final Natufian). (MP4)

S3 Movie Rotating 3D photogrammetry (front view) of the clay zoomorphic figurine EM99 7121 (Fig 4B – Final Natufian). (MP4)

S4 Movie Rotating 3D photogrammetry (right profile view) of the clay zoomorphic figurine EM99 7121 (Fig 4B – Final Natufian). (MP4)

S5 Movie Rotating 3D photogrammetry (right profile view) of the clay anthropomorphic figurine EM75 4551.17 (Fig 4C – Early Natufian). (MP4)

S6 Movie Rotating 3D photogrammetry (top-back view) of the clay anthropomorphic figurine EM75 4551.17 (Fig 4C – Early Natufian). (MP4)

S7 Movie Rotating 3D photogrammetry (front view) of the clay anthropomorphic figurine EM01 8745.6 (Fig 4D – Final Natufian). (MP4)

S8 Movie Rotating 3D photogrammetry (right profile view) of the clay ornithomorphic figurine EM97 6143 (Fig 4E – Final Natufian). (MP4)

S9 Movie Rotating 3D photogrammetry (right profile view) of the clay ornithomorphic figurine EM74 3339 (Fig 4J – Early Natufian). (MP4)

S10 Movie Rotating 3D photogrammetry (left profile view) of the clay ornithomorphic figurine HT86 1632 (Fig 4M – Late Natufian). (MP4)

S11 Movie Rotating 3D photogrammetry (right profile view) of the clay ornithomorphic figurine HT86 1272 (Fig 4N – Late Natufian). (MP4)
